# Sphingosine‐1‐Phosphate Receptor 3 Confers Tumor Metastasis in Lung Cancer Resistant to Third‐Generation EGFR Inhibitor

**DOI:** 10.1002/mco2.70744

**Published:** 2026-04-23

**Authors:** Mengzhen Lai, Jiaying Chen, Ye Qin, Hui Zhang, Zilu Pan, Tao Zhang, Linjiang Tong, Haotian Tang, Gang Bai, Qiupei Liu, Yan Li, Fang Feng, Peiran Song, Yingqiang Liu, Yi Chen, Yan Fang, Bencan Tang, Meiyu Geng, Ker Yu, Hao Chen, Jian Ding, Hua Xie

**Affiliations:** ^1^ Division of Antitumor Pharmacology & State Key Laboratory of Drug Research Shanghai Institute of Materia Medica Chinese Academy of Sciences Shanghai China; ^2^ Department of Pharmacology School of Pharmacy Fudan University Shanghai China; ^3^ University of Chinese Academy of Sciences Beijing China; ^4^ State Key Laboratory of Chemical Biology Molecular Imaging Center Shanghai Institute of Materia Medica Chinese Academy of Sciences Shanghai China; ^5^ Shanghai Lung Cancer Center Shanghai Chest Hospital Shanghai Jiao Tong University School of Medicine Shanghai China; ^6^ Zhongshan Institute for Drug Discovery Shanghai Institute of Materia Medica Chinese Academy of Sciences Zhongshan China; ^7^ Key Laboratory for Carbonaceous Waste Processing and Process Intensification Research of Zhejiang Province Department of Chemical and Environmental Engineering The University of Nottingham Ningbo China Ningbo China

**Keywords:** EGFR inhibitor, EMT, lung cancer, metastasis, S1PR3

## Abstract

The third‐generation EGFR tyrosine kinase inhibitor (TKI) osimertinib (AZD9291) has significantly improved the survival in EGFR‐mutant lung cancer patients. Our team developed limertinib (ASK120067), a novel third‐generation EGFR inhibitor with remarkable antitumor effects, which has been launched in China. Despite initial therapeutic responses, EGFR TKIs‐treated patients ultimately experience fatal metastatic recurrence and disease progression. However, the underlying mechanism of driving metastasis remains poorly understood. Here, we aim to investigate the pro‐metastatic mechanism following treatment with third‐generation EGFR TKIs. Transcriptomics analyses of EGFR TKI‐resistant tumor models revealed an aberrant upregulation of S1PR3, which conferred enhanced metastatic potential to lung cancer. S1PR3 inhibition dramatically reduced metastasis in resistant cells, while its overexpression potentiated metastatic abilities in parental cells. Notably, S1PR3 was highly enriched in clinical samples with AZD9291 resistance and correlates with poor prognosis. Mechanistically, we found that S1PR3 upregulated RAC1‐GTP expression to activate PAK1, thereby promoting epithelial‐mesenchymal transition (EMT) and enhancing metastatic capacity of resistant cells. Further studies identified that the overexpression of fibroblast growth factor receptor 1 (FGFR1) increased S1PR3 expression through signal transducer and activator of transcription 4 (STAT4) to promote the emergence of metastatic‐resistant cells. Importantly, targeting S1PR3 or FGFR1 blocks metastasis in EGFR TKI‐resistant models.

## Graphical abstract

• S1PR3 drives metastasis in EGFR TKI‐resistant lung cancer by activating RAC1‐PAK1 signaling and promoting EMT.

• FGFR1‐STAT4 axis upregulates S1PR3, enabling metastatic escape in resistant tumors.

• Targeting S1PR3 or FGFR1 suppresses metastasis, offering a therapeutic strategy for EGFR TKI‐resistant lung cancer.

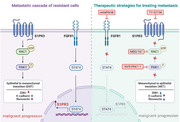

## 1 Introduction

Lung cancer remains the leading cause of cancer‐related mortality globally, accounting for more than 90% of cancer‐related deaths due to its high metastatic potential [1, 2, 3, 4]. Epidermal growth factor receptor (EGFR) is a classical target for lung cancer treatment, with several inhibitors already approved [5]. Osimertinib (AZD9291) is the first approved third‐generation EGFR tyrosine kinase inhibitor (TKI) with striking efficacy in lung cancer patients [6, 7]. Our previous work introduced ASK120067 (abbreviated as 120067), a novel third‐generation EGFR inhibitor with comparable antitumor activity to AZD9291, which was launched in China in 2025 [8]. Lung cancer patients with brain metastasis respond well to these third‐generation EGFR TKIs due to its potent efficacy and high blood–brain barrier (BBB) penetrance. However, its therapeutic response is not durable, and metastatic relapses constitute the primary cause of treatment failure in EGFR TKIs‐treated lung cancer patients [9, 10, 11, 12]. Emerging evidence demonstrates that cancer cells resisting therapy are vulnerable to retaining or acquiring migration and invasion abilities, further complicating disease management [13, 14].

Recent studies have revealed that elevated expression of S100A9 is associated with lethal metastatic relapse in lung cancer patients following AZD9291 treatment [15]. Furthermore, research has shown that PSAT1 overexpression promotes tumor metastasis in lung cancer, leading to resistance to erlotinib, the first‐generation EGFR inhibitor [16]. Moreover, it has been proved that the upregulation of integrin α5 contributed to the enhancement of migration and invasion ability of lung cancer cells resistant to icotinib, a second‐generation EGFR inhibitor [14]. Nevertheless, the mechanisms associated with metastatic relapse in lung cancer patients after EGFR TKIs resistance remain insufficiently understood.

G protein‐coupled receptors (GPCRs), the largest membrane proteins family encoded by the human genome and represent one of the most successful therapeutic targets in oncology. GPCRs are broadly classified into several subfamilies, including Rhodopsin, Secretin, Adhesion, Glutamate, and Frizzled, which play critical roles in tumorigenesis and metastasis. For instance, CXCR4 promotes tumor cell migration and proliferation and is highly expressed in various solid tumors and hematological malignancies [17]. LGR5 activates canonical Wnt/β‐catenin signaling to mediate cell–cell adhesion and is markedly upregulated in gastrointestinal cancers [18, 19]. Similarly, CCR7, commonly upregulated in multiple cancer types, facilitates tumor cell migration and metastatic dissemination through its chemotactic functions [20, 21].

Among these clinically relevant GPCRs, sphingosine‐1‐phosphate receptor 3 (S1PR3) has emerged as a particularly promising yet understudied therapeutic target [22, 23, 24]. As a critical member of the GPCR family, S1PR3 binds to its ligand S1P, a bioactive phospholipid, and plays a key role in cell proliferation, survival, and metastasis by activating downstream signaling, predominantly including PI3K‐AKT, RAS‐RAF‐ERK, RAC1‐PAK1, and Rho‐ROCK‐NF‐κB pathway [25]. Increased expression of S1PR3 has been reported to be associated with cancer progression in various malignancies, including lung cancer and breast cancer [26, 27, 28]. However, studies that have been conducted on the functional role of S1PR3 in tumor progression are relatively limited, and the mechanism of S1PR3 mediates metastasis in EGFR TKI‐resistant tumors has not yet been reported.

In this study, we demonstrated that S1PR3 is the crucial molecule that mediated metastasis in EGFR TKI‐resistant tumors, with its expression markedly upregulated in preclinical EGFR TKI resistance models and clinical tumor samples. We also confirmed that S1PR3 promotes metastasis via the RAC1‐PAK1 pathway rather than other pathways. Furthermore, FGFR1 transcriptionally regulates S1PR3 overexpression through the transcription factor STAT4. Our findings uncovered the clinical relevance of S1PR3 in resistant lung cancer and that targeting S1PR3 could be a promising therapeutic strategy to reduce metastasis in third‐generation EGFR TKIs‐resistant lung cancer.

## 2 Results

### 2.1 S1PR3 is a Key Mediator of Metastasis in EGFR Inhibitor‐Resistant Lung Cancer Cells

**FIGURE 1:**
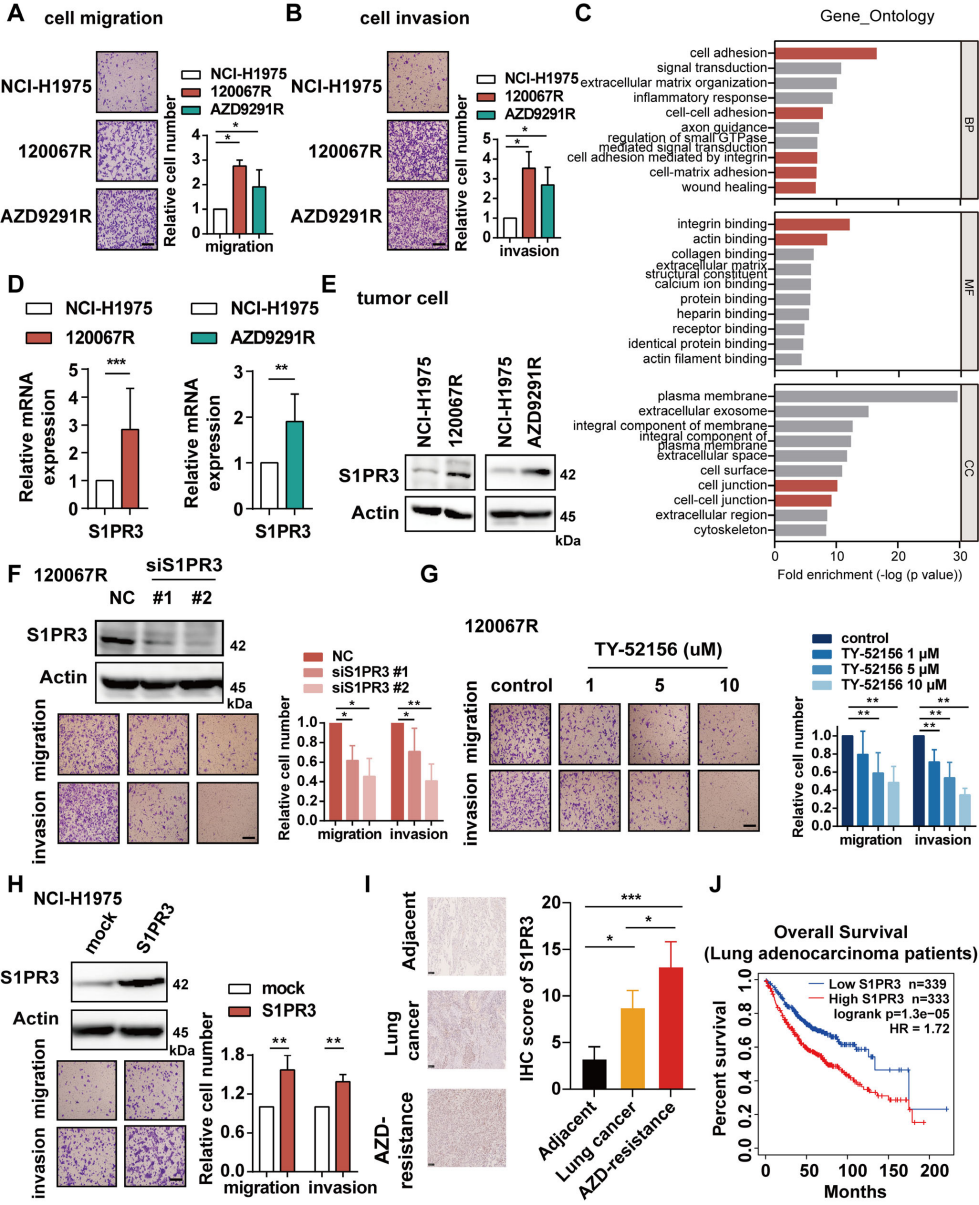
S1PR3 is a key mediator of metastasis in EGFR inhibitor‐resistant lung cancer cells. (A) Migration or (B) invasion of parental cells (NCI‐H1975) and resistant cells (120067R and AZD9291R) detected by trans‐well assays. (C) GO enrichment analysis of differentially expressed genes (fold change ≥ 2) between parental and resistant cells as identified through RNA‐seq analysis. (D) Gene or (E) protein expression of S1PR3 in parental cells and resistant cells was analyzed by RT‐PCR assay or immunoblotting. (F) Migration and invasion of 120067R cells after siS1PR3 transfection or (G) treated with S1PR3 inhibitor TY‐52156. (H) Migration and invasion of NCI‐H1975 cells exogenous overexpressed S1PR3. (I) IHC staining of S1PR3 on human tissue specimens from adjacent normal tissues, lung cancer, and AZD9291‐resistant lung cancer. (J) Overall survival for lung adenocarcinoma patients with high or low S1PR3 expression was analyzed by Kaplan–Meier survival analysis. Scale bar, 100 µm. *p < 0.05, ** *p* < 0.01, ***p < 0.001.

Our previous work introduced ASK120067 (abbreviated as 120067), a novel third‐generation EGFR inhibitor, which has been launched in China, with comparable preclinical and clinical antitumor activities to AZD9291. We generated ASK120067‐resistant cells (120067R) and AZD9291‐resistant cells (AZD9291R) by culturing NCI‐H1975 cells with gradually increasing concentrations of 120067 or AZD9291, respectively, until resistant variants emerged. Cell proliferation assays demonstrated that parental NCI‐H1975 cells remained highly sensitive to both ASK120067 (IC_50_ = 0.03 µM) and AZD9291 (IC_50_ = 0.05 µM), whereas the 120067R and AZD9291R resistant variants exhibited markedly reduced drug sensitivity, with IC_50_ values increasing to 5.38 and 7.19 µM, respectively, demonstrating a more than 100‐fold increase in resistance compared to parental cells (Figure S1A). Whole genome sequencing (WGS) results from our previous study confirmed the original EGFR L858R/T790M mutation was maintained at lower levels in both resistant cells compared to parental NCI‐H1975 cells, which is consistent with the clinical observation of EGFR T790M reduction or disappearance following AZD9291 treatment [29, 30]. Notably, no acquired mutations in EGFR, including C797S mutation, were found in either resistant cell lines [8]. Interestingly, we noticed that parental NCI‐H1975 cells presented round or short fusiform morphology, whereas 120067R‐ and AZD9291R‐resistant cells exhibited spindle‐like morphology (Figure S1B). Furthermore, H&E staining in tumor tissues showed similar results in line with the in vitro findings (Figure S1C). These results indicated that resistant cells might acquire an enhanced metastatic phenotype compared to parental cells. To investigate this possibility, we assessed the metastatic ability of parental and resistant cells using trans‐well assay and observed that both 120067R‐ and AZD9291R‐resistant cells exhibited enhanced migration and invasion abilities compared to parental NCI‐H1975 cells (Figure 1A,B). These data demonstrated that EGFR TKI‐resistant cells exhibited more invasive and migrative phenotypes relative to parental cells.

To uncover the underlying mechanism involved in an enhanced metastasis phenotype in resistant cells, we performed Gene Ontology (GO) enrichment analysis on RNA‐seq expression data derived from parental and resistant cells. The analysis revealed an enrichment of tumor metastasis‐related signatures, such as cell adhesion and integrin binding, which were enriched in resistant cells (Figure 1C). Recognizing the critical role of GPCR signaling in cancer metastasis, we next sought to identify potentially druggable GPCR targets within these metastasis‐associated pathways. By intersecting the GO‐derived metastasis‐related gene signatures with the GPCR genes from GPCRdb, we identified seven genes that were concurrently classified as both tumor metastasis‐related genes and canonical GPCR genes (Figure S1D and Table S1). Among these candidates, S1PR3 was uniquely and consistently upregulated across both 120067R‐ and AZD9291R‐resistant cell models. Given the tumor‐promoting mechanisms of S1PR3 remain incompletely elucidated, we sought to systematically investigate its specific role in mediating the metastatic phenotype of resistant cells [26]. Consistent with the RNA‐seq data, both mRNA and protein levels of S1PR3 were elevated in 120067R and AZD9291R cells compared to NCI‐H1975 cells (Figure 1D,E), and the protein expression of S1PR3 in 120067R or AZD9291R xenograft tumors was upregulated (Figure S1E).

To determine the role of S1PR3 in metastasis of the resistant cells, we knocked down S1PR3 with siRNAs in 120067R cells and found that downregulation of S1PR3 expression significantly decreased cell migration and invasion (Figure 1F). In addition, treatment with S1PR3 selective inhibitor TY‐52156 also dose dependently blocked the migration and invasion of 120067R cells compared to the control group (Figure 1G). Consistent with the findings in 120067R cells, cell migration and invasion were suppressed by interfering S1PR3 in AZD9291R cells (Figure S1F). Moreover, exogenous overexpression of S1PR3 in NCI‐H1975 cells enhanced migration and invasion abilities (Figure 1H). These data demonstrated that S1PR3 is a critical regulator of metastasis in EGFR inhibitor‐resistant cells.

To validate the clinical relevance, we examined S1PR3 expression in primary and AZD9291‐resistant tumors obtained from lung cancer patients. Immunohistochemistry (IHC) staining revealed that the protein level of S1PR3 was elevated in tumor specimens compared to adjacent tissue. Notably, S1PR3 expression was further increased in AZD9291‐resistant lung cancer tissue specimens compared to primary tumor tissues, supporting its association with EGFR TKI‐resistant progression in this patient‐derived cohort (Figure 1I). Kaplan–Meier survival analysis from the TCGA database (primarily untreated primary lung adenocarcinoma cases) showed that high expression of S1PR3 resulted in a poor overall survival (Figure 1J). These clinical findings supported a positive correlation of S1PR3 with lung cancer progression.

### 2.2 S1PR3 Contributes to the High Metastatic Potency of EGFR Inhibitor‐Resistant Lung Cancer Cells Through the RAC1‐PAK1 Signaling Pathway

**FIGURE 2:**
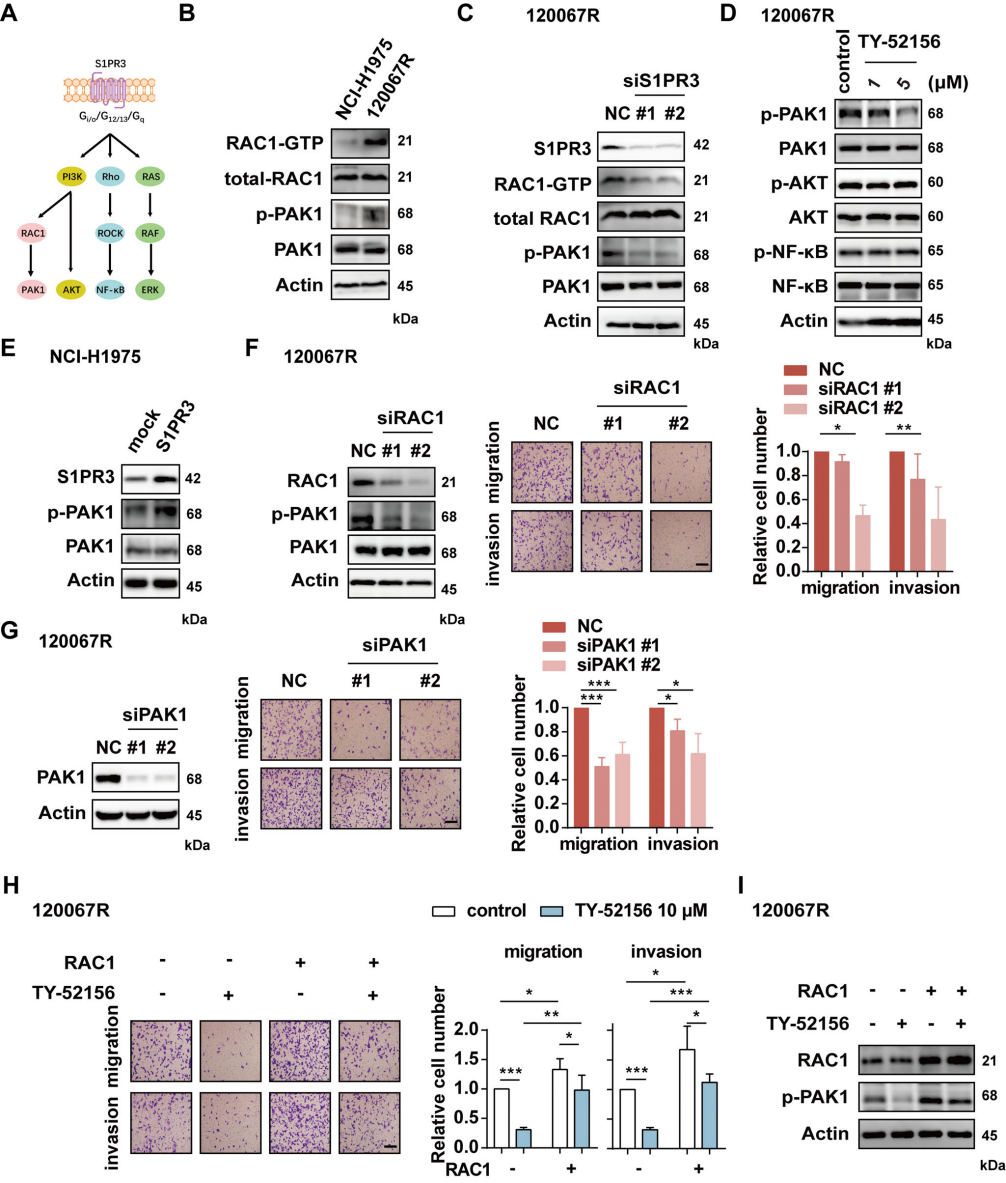
S1PR3 contributed to the metastasis in EGFR TKIs‐resistant cells through the RAC1‐PAK1 signaling pathway. (A) Schematic diagram of S1PR3 downstream pathways. (B) The protein expression of NCI‐H1975 cells and 120067R cells was detected by immunoblotting. (C) Immunoblotting on 120067R cells after S1PR3 knockdown or (D) treated with S1PR3 inhibitor TY‐52156. (E) Immunoblotting of NCI‐H1975 cells showed exogenous overexpression of S1PR3. (F) Migration and invasion of 120067R cells after RAC1 or (G) PAK1 knockdown. (H) Migration and invasion or (I) immunoblotting of 120067R cells showed exogenous overexpression of RAC1 and subsequently treated with S1PR3 inhibitor. Scale bar, 100 µm. *p < 0.05, **p < 0.01, *** *p* < 0.001.

Given that S1PR3 exerts pro‐tumorigenic effects through multiple pathways, including RAC1‐PAK1, PI3K‐AKT, Rho‐ROCK‐NF‐κB, and RAS‐RAF‐ERK pathways (Figure 2A) [25], we sought to determine which pathway plays a key role in mediating metastasis in EGFR TKIs‐resistant tumors. Subsequently, we analyzed the activation level of the aforementioned signaling pathways in resistant cells. Immunoblotting results showed a significant increase in RAC1‐PAK1, p‐AKT, and p‐NF‐κB in 120067R cells compared to NCI‐H1975 cells, while the expression of p‐ERK remained unchanged (Figure 2B and Figure S2A). These findings were consistent with the 120067R xenograft model as well (Figure S2B). We further examined the downstream effects of S1PR3 signaling by transfecting 120067R cells with siRNAs targeting S1PR3. The results revealed that only the activation of RAC1 and PAK1 was reversed, while AKT and NF‐κB pathway activation remained unaffected (Figure 2C and Figure S2C). Treatment with S1PR3 inhibitor TY‐52156 also resulted in a dose‐dependent decrease in phosphorylated PAK1 levels without affecting AKT or NF‐κB phosphorylation (Figure 2D). Moreover, exogenous overexpression of S1PR3 in NCI‐H1975 cells upregulated the phosphorylation level of PAK1 (Figure 2E). These findings suggested that RAC1‐PAK1 represented a key downstream signaling axis of S1PR3 in resistant cells.

To determine whether RAC1 promotes migration and invasion of 120067R cells, we interfered RAC1 with siRNA or treated with RAC1 inhibitor MBQ‐167 and found a decrease in migration and invasion (Figure 2F and Figure S2D). Overexpression of RAC1 enhanced the migration and invasion ability of NCI‐H1975 cells (Figure S2E). We then investigated whether the activation of PAK1 is regulated by RAC1 and found that the phosphorylation level of PAK1 was abolished by two different RAC1 siRNA in 120067R cells (Figure 2F). As expected, overexpression of RAC1 promoted the activation of PAK1 in NCI‐H1975 cells (Figure S2E). Likewise, depletion of PAK1 or treatment with PAK1 inhibitor NVS‐PAK1‐1 effectively blocked migration and invasion of 120067R cells (Figure 2G and Figure S2F), and PAK1 overexpression facilitated migration and invasion in NCI‐H1975 cells (Figure S2G).

To elucidate whether RAC1 is involved in S1PR3‐mediated metastasis, we overexpressed RAC1 in 120067R cells and subsequently treated the cells with S1PR3 inhibitor TY‐52156. The data demonstrated that RAC1‐induced enhancement of cell migration and invasion was significantly suppressed by the inhibitor TY‐52156. Notably, the inhibitory effect of TY‐52156 could be partially rescued by RAC1 overexpression (Figure 2H). Immunoblotting data confirmed that overexpression of RAC1 in 120067R cells further induced the PAK1 phosphorylation, and this effect was reversed by S1PR3 inhibitor TY‐52156 (Figure 2I). These results discovered that upregulation of the S1PR3‐RAC1‐PAK1 axis was required for migration and invasion in EGFR inhibitor‐resistant cells.

### 2.3 S1PR3 Expression Correlated With EMT Progress in EGFR Inhibitor‐Resistant Cells, and S1PR3 Silencing Reversed EMT Through the RAC1‐PAK1 Signaling Pathway

**FIGURE 3:**
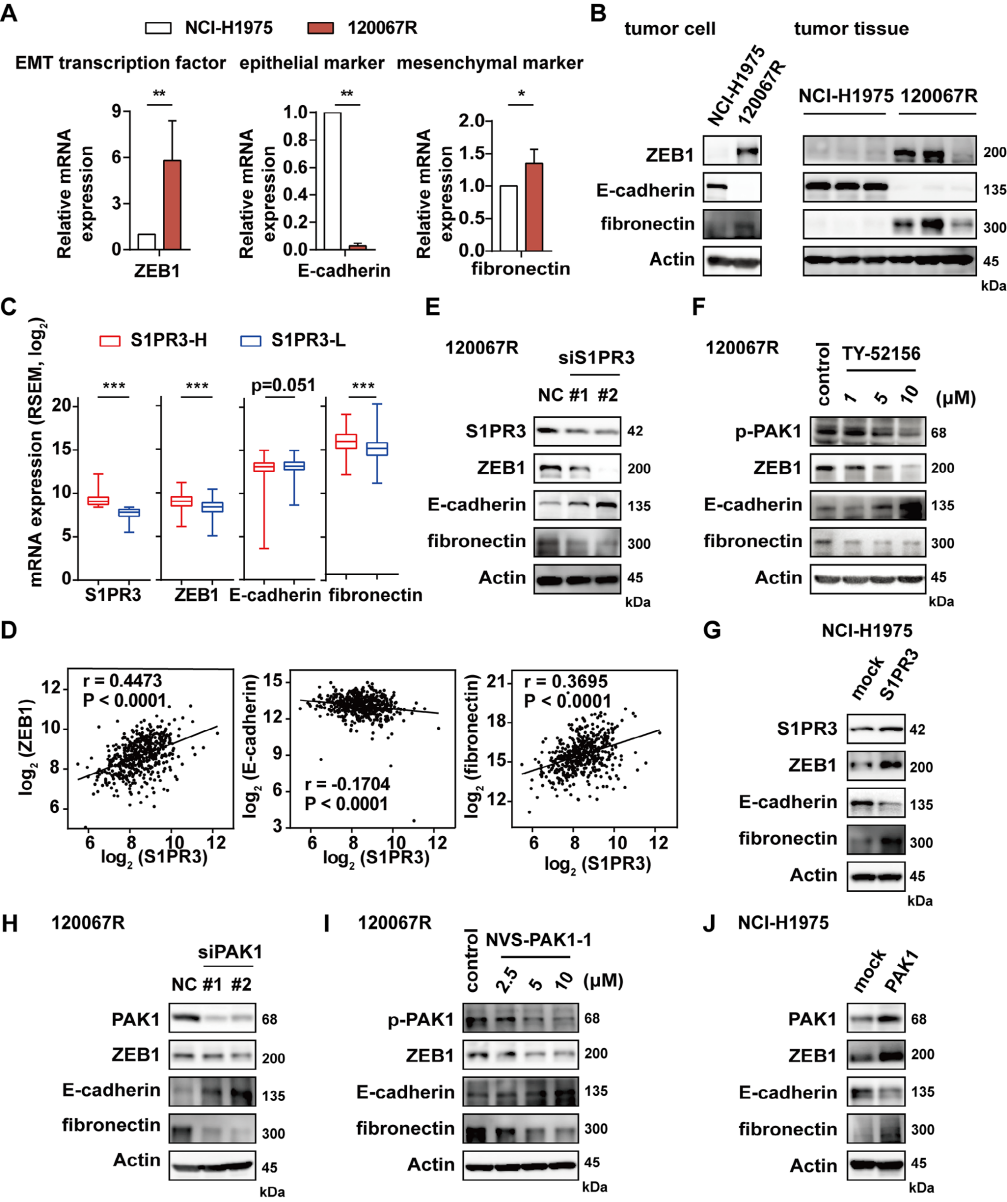
S1PR3‐RAC1‐PAK1 axis is associated with the EMT progression of resistant cells. (A) Gene or (B) protein expression of EMT markers detected by RT‐PCR assays and immunoblotting. (C) Transcriptome signature of EMT in S1PR3^high^ group (S1PR3‐H) and S1PR3^low^ group (S1PR3‐L) of lung adenocarcinoma patients. (D) The association between S1PR3 and EMT‐related genes level in lung adenocarcinoma patients. (E) Immunoblotting on 120067R cells after S1PR3 knockdown or (F) treatment with S1PR3 inhibitor. (G) Immunoblotting of NCI‐H1975 cells showed exogenous overexpression of S1PR3. (H) Immunoblotting on 120067R cells after PAK1 knockdown or (I) treatment with PAK1 inhibitor. (J) Immunoblotting data of NCI‐H1975 cells showed exogenous overexpression of PAK1. *p < 0.05, **p < 0.01, *** *p* < 0.001.

Numerous studies have reported that EMT is closely linked to cancer metastasis [31, 32]. The above findings showed that resistant cells obtained changes in cell morphology and metastatic potency compared to parental cells, leading us to question whether EMT occurs in resistant cells. We subsequently analyzed the expression of EMT markers. RT‐PCR analysis results showed an increase in the expression of EMT transcription factors (ZEB1 and ZEB2) and mesenchymal markers (fibronectin, N‐cadherin, and vimentin), accompanied by decreased expression of epithelial markers (E‐cadherin and occludin) in 120067R cells relative to NCI‐H1975 cells (Figure 3A and Figure S3A). In line with the expression of mRNA, immunoblotting confirmed the upregulation of ZEB1 and fibronectin, alongside the decrease of E‐cadherin in 120067R cells and tumor tissues (Figure 3B). Similar results were obtained in AZD9291R cells and tumor tissues (Figure S3B). To evaluate whether EMT was involved in 120067R cell migration, we found that silencing ZEB1 in 120067R cells markedly impaired cell migration and invasion, accompanied by upregulation of E‐cadherin and downregulation of fibronectin, indicating that EMT was closely associated with 120067R cells migration (Figure S3C).

We further verified whether the expression of S1PR3 was associated with EMT‐related genes in a lung adenocarcinoma cohort (503 patients) from the TCGA database. As shown in Figure 3C, the mRNA level of S1PR3 was positively associated with ZEB1 and fibronectin expression but negatively associated with E‐cadherin expression. The expression of S1PR3 was also positively correlated with ZEB2, N‐cadherin, and vimentin expression, while negatively correlated with occludin expression (Figure S3D). Likewise, similar conclusions were drawn from the correlation analysis (Figure 3D and Figure S3E). These results indicated that the expression of S1PR3 showed a positive correlation with EMT‐related genes.

To further illustrate the correlation between the expression of S1PR3 and EMT‐related protein, we tested whether S1PR3 interference or inhibition in resistant cells could reverse EMT. S1PR3 interference in 120067R cells and AZD9291R cells induced mesenchymal‐epithelial transition (MET), as shown by the upregulation of E‐cadherin, as well as the downregulation of ZEB1 and fibronectin (Figure 3E and Figure S3F). Consistent results were also obtained for S1PR3 inhibitor TY‐52156 treatment in 120067R cells (Figure 3F). In addition, S1PR3 overexpression in NCI‐H1975 cells could further promote EMT progress (Figure 3G). Likewise, interference or treatment with downstream effectors RAC1 and PAK1 inhibitors in 120067R cells resulted in MET (Figure S3G,H and Figure 3H,I). Moreover, overexpression of RAC1 or PAK1 significantly induced EMT in NCI‐H1975 cells (Figure S3I and Figure 3J). These data collectively suggested that the S1PR3‐RAC1‐PAK1 signaling axis promotes EGFR inhibitor‐resistant cell migration and invasion by regulating the EMT process.

### 2.4 FGFR1 Upregulation Promotes S1PR3 Expression and Mediated Metastasis in EGFR TKI‐Resistant Lung Cancer Cells

**FIGURE 4:**
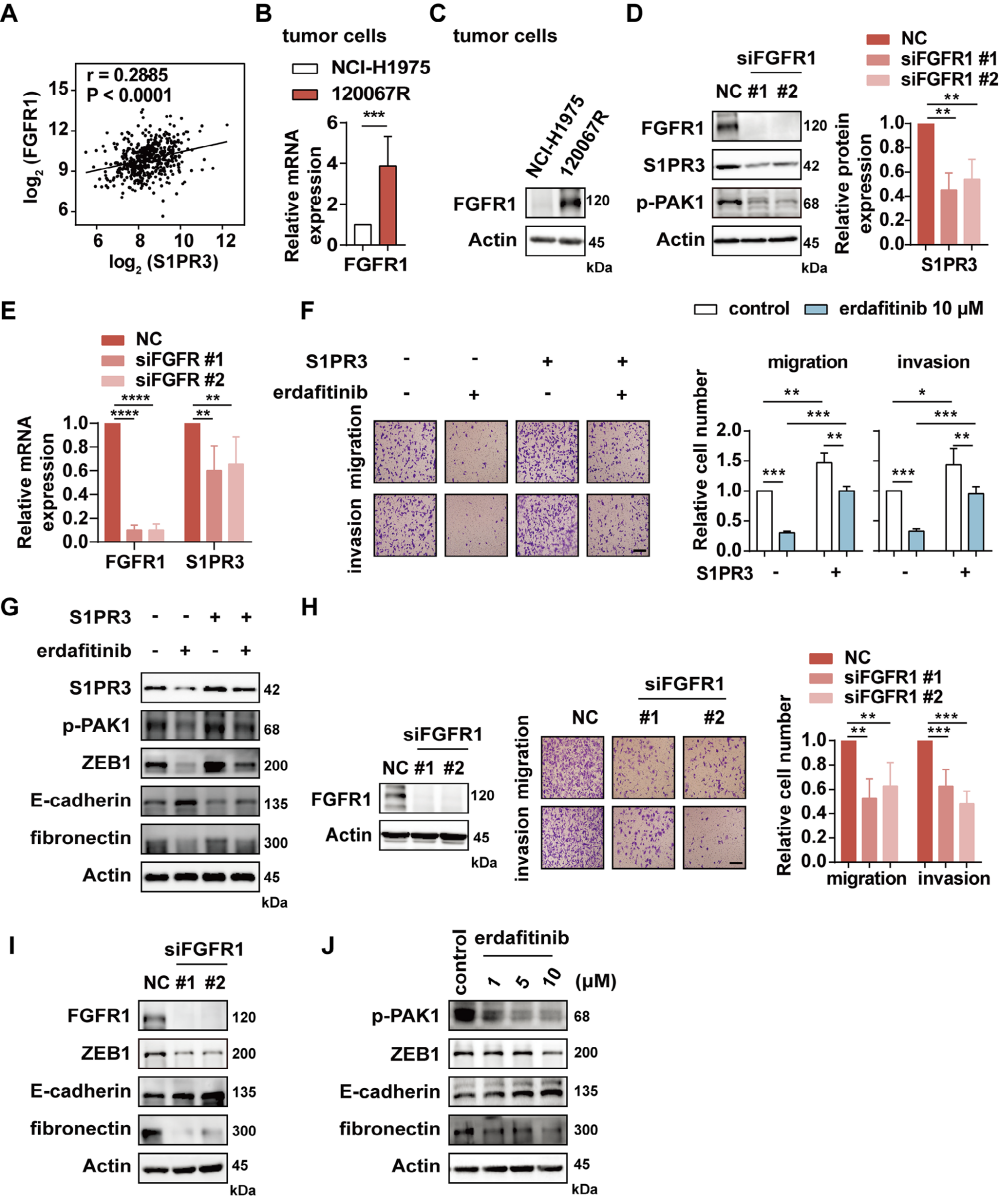
FGFR1 regulates S1PR3 transcription and promotes EGFR TKI‐resistant cell metastasis. (A) The association between S1PR3 and FGFR1 levels in lung adenocarcinoma patients from TCGA database. (B) Gene or (C) protein expression of FGFR1 in parental and resistant cells. (D) Protein or (E) genes expression of 120067R cells after FGFR1 knockdown. (F) Migration and invasion or (G) immunoblotting of 120067R cells showed exogenous overexpression of S1PR3 and subsequently treated with FGFR1 inhibitor. (H) Migration and invasion of 120067R cells after FGFR1 knockdown. (I) Immunoblotting results of 120067R cells after siFGFR1 transfection or (J) treatment with FGFR1 inhibitor. Scale bar, 100 µm. *p < 0.05, **p < 0.01, *** *p* < 0.001.

The aforementioned data confirmed that S1PR3 played a key role in EGFR TKI‐resistant lung cancer cell metastasis. To explore the potential molecular mechanisms underlying the upregulation of S1PR3, RNA‐seq data revealed that fibroblast growth factor receptor 1 (FGFR1) was upregulated in 120067R cells compared to the parental NCI‐H1975 cells (Figure S4A). Given that previous studies have reported an association between FGFR1 overexpression and tumor metastasis [33, 34, 35, 36], we further examined the relationship between FGFR1 and S1PR3. TCGA database analysis in lung adenocarcinoma patients confirmed a positive correlation between FGFR1 and S1PR3 expression (Figure 4A). Based on these findings, we herein hypothesized that FGFR1 might contribute to the increased S1PR3 expression in 120067R‐resistant cells.

To validate this hypothesis, we detected the expression of FGFR1 in both parental and resistant cells. The data showed that the mRNA and protein level of FGFR1 was consistently upregulated in 120067R cells (Figure 4B,C). To uncover whether FGFR1 regulates S1PR3 expression, we treated 120067R cells with siRNAs targeting FGFR1 and observed decreased abundance of S1PR3, as well as the downstream signaling phosphorylation of PAK1 level (Figure 4D). Moreover, FGFR1 depletion resulted in reduced S1PR3 mRNA levels in 120067R cells (Figure 4E), suggesting that FGFR1 transcriptionally regulated the expression of S1PR3. To further confirm that FGFR1 mediated metastasis by regulating S1PR3 expression, we overexpressed S1PR3 in 120067R cells and then treated with the FGFR1 inhibitor erdafitinib. S1PR3 overexpression markedly induced migration and invasion, and the effect was remarkably reversed after treatment with the FGFR1 inhibitor erdafitinib. Notably, the inhibitory effect of erdafitinib could be partially rescued by S1PR3 overexpression (Figure 4F). Immunoblotting analysis revealed that overexpression of S1PR3 in 120067R cells further induced EMT and increased phosphorylated PAK1 levels, both of which were reversed by the FGFR1 inhibitor (Figure 4G).

Furthermore, we explored whether FGFR1 is involved in 120067R cell metastasis and found that FGFR1 knockdown or erdafitinib treatment remarkably decreased the migration and invasion of 120067R cells (Figure 4H and Figure S4B). Immunoblotting results confirmed that interfering with FGFR1 or treatment with FGFR1 inhibitor reversed EMT (Figure 4I,J). These results indicated that the promoting function of S1PR3 in 120067R cells metastasis was mediated by increased expression of FGFR1 at the transcriptional level.

### 2.5 FGFR1 Contributed to the Upregulated of S1PR3 by Regulating the Transcription Factor STAT4 in EGFR TKI‐Resistant Lung Cancer Cells

**FIGURE 5:**
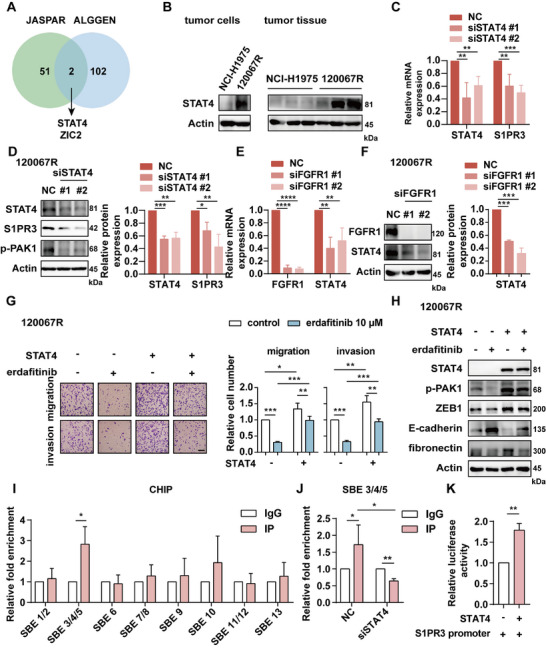
FGFR1 regulates S1PR3 transcription by regulating the transcription factor STAT4. (A) The Venn diagram of transcription factors binds to the promoter of S1PR3 predicted in the JASPAR database and ALGGEN PROMO database. (B) Immunoblotting results. (C) Gene or (D) protein expression in 120067R cells after STAT4 knockdown. (E) Gene or (F) protein expression in 120067R cells after FGFR1 knockdown. (G) Migration and invasion or (H) immunoblotting of 120067R cells showed exogenous overexpression of STAT4 and subsequently treated with FGFR1 inhibitor. (I) STAT4 ChIP‐qPCR of the S1PR3 promoter in 120067R cells. (J) ChIP‐qPCR analysis in 120067R cells after STAT4 knockdown. (K) Relative luciferase activity of HEK293T cells co‐transfection of STAT4 and human S1PR3 promotor. Scale bar, 100 µm. *p < 0.05, **p < 0.01, *** *p* < 0.001, **** *p* < 0.0001.

Since the regulation of S1PR3 expression by FGFR1 occurred at the transcriptional level, we sought to identify transcriptional regulators of S1PR3. JASPAR database (https://jaspar.genereg.net/) and ALGGEN PROMO database (http://alggen.lsi.upc.es/) were used to predict transcription factors that bind the promoter of S1PR3. We obtained a total of 53 and 104 potential transcription factors in JASPAR and ALGGEN PROMO databases, respectively (Table S2). A Veen diagram was constructed to display the overlapping transcriptional factors among these two databases (Figure 5A). The transcription factors predicted in both databases include signal transducer and activator of transcription 4 (STAT4) and Zic family member 2 (ZIC2). It has been reported that STATs are downstream transcriptional targets of FGFRs and are involved in cancer metastasis [37], whereas ZIC2 is mainly transcriptional regulation in the nervous system [38]. We observed that STAT4 expression was increased in 120067R cells and tumor tissues (Figure 5B). We thus speculated that STAT4 might be the key transcription factor that regulated S1PR3 expression.

Consistent with the predicted consequence, silencing STAT4 decreased the expression of S1PR3 at both mRNA and protein levels (Figure 5C,D). Then, we sought to address whether FGFR1 silencing reduced the expression of STAT4 and observed that FGFR1 interference in 120067R cells suppressed the expression of STAT4 at both mRNA and protein levels (Figure 5E,F). Moreover, STAT4‐silenced 120067R cells showed less migrative and invasive effects (Figure S5A). To explore the role of STAT4 in metastasis regulated by FGFR1, we overexpressed STAT4 in 120067R cells and then treated them with FGFR1 inhibitor erdafitinib. STAT4 overexpression promoted migration and invasion in 120067R cells, while the ability of metastasis could be impaired by erdafitinib (Figure 5G). Immunoblotting analysis revealed that STAT4 overexpression further induced EMT in 120067R cells, whereas the EMT progress could be reversed by FGFR1 inhibitor (Figure 5H). These results demonstrated that STAT4 played a key role in the regulation of S1PR3 expression by FGFR1.

To identify that STAT4 could directly bind to the S1PR3 promoter, a ChIP‐qPCR assay was performed. Compared with non‐specific IgG antibody, the promoter of the putative S1PR3‐binding site (SEB3/4/5) showed a threefold enrichment after immunoprecipitation with an anti‐STAT4 antibody in 120067R cells. At the same time, no significant differences were observed at other binding sites (Figure 5I). Furthermore, transfection with STAT4 siRNAs could reduce the binding of STAT4 and S1PR3 promoters (Figure 5J). To further confirm the regulatory role of STAT4 on S1PR3 expression, we performed promoter luciferase reporter assay to test the effect of overexpression of STAT4 and S1PR3 promoter in HEK293T cells. We found that the luciferase activity was upregulated after overexpressing STAT4 in the presence of S1PR3 promoter, indicating that STAT4 could promote the transcriptional level of S1PR3 (Figure 5K). These results revealed that FGFR1‐STAT4‐S1PR3 axis was a critical regulatory pathway driving metastasis in 120067R cells.

To systematically evaluate the generalizability of FGFR1‐STAT4‐S1PR3‐PAK1 axis across different cell models resistant to third‐generation EGFR‐TKIs, we developed resistant variants of PC‐9 cells, another third‐generation EGFR‐TKI sensitive cell line, through gradual exposure to increasing concentrations of 120067 or AZD9291. Cell proliferation assays revealed that both 120067‐resistant cells (PC‐9‐67R) and AZD9291‐resistant cells (PC‐9‐AZDR) exhibited profound acquired resistance, with IC_50_ values shifting from nanomolar to micromolar ranges, corresponding to over 200‐fold resistance (Figure S5B). Notably, consistent with our findings in NCI‐H1975‐resistant cell models, Western blot analysis demonstrated elevated expression of FGFR1, S1PR3, STAT4, and p‐PAK1 in both PC‐9‐derived resistant lines (Figure S5C), confirming the broad relevance of the FGFR1‐STAT4‐S1PR3‐PAK1 axis across distinct EGFR‐TKI‐resistant models.

### 2.6 Targeting Inhibition FGFR1 or S1PR3 to Suppress Metastasis in Animal Models of EGFR TKI‐Resistant Lung Cancer

**FIGURE 6:**
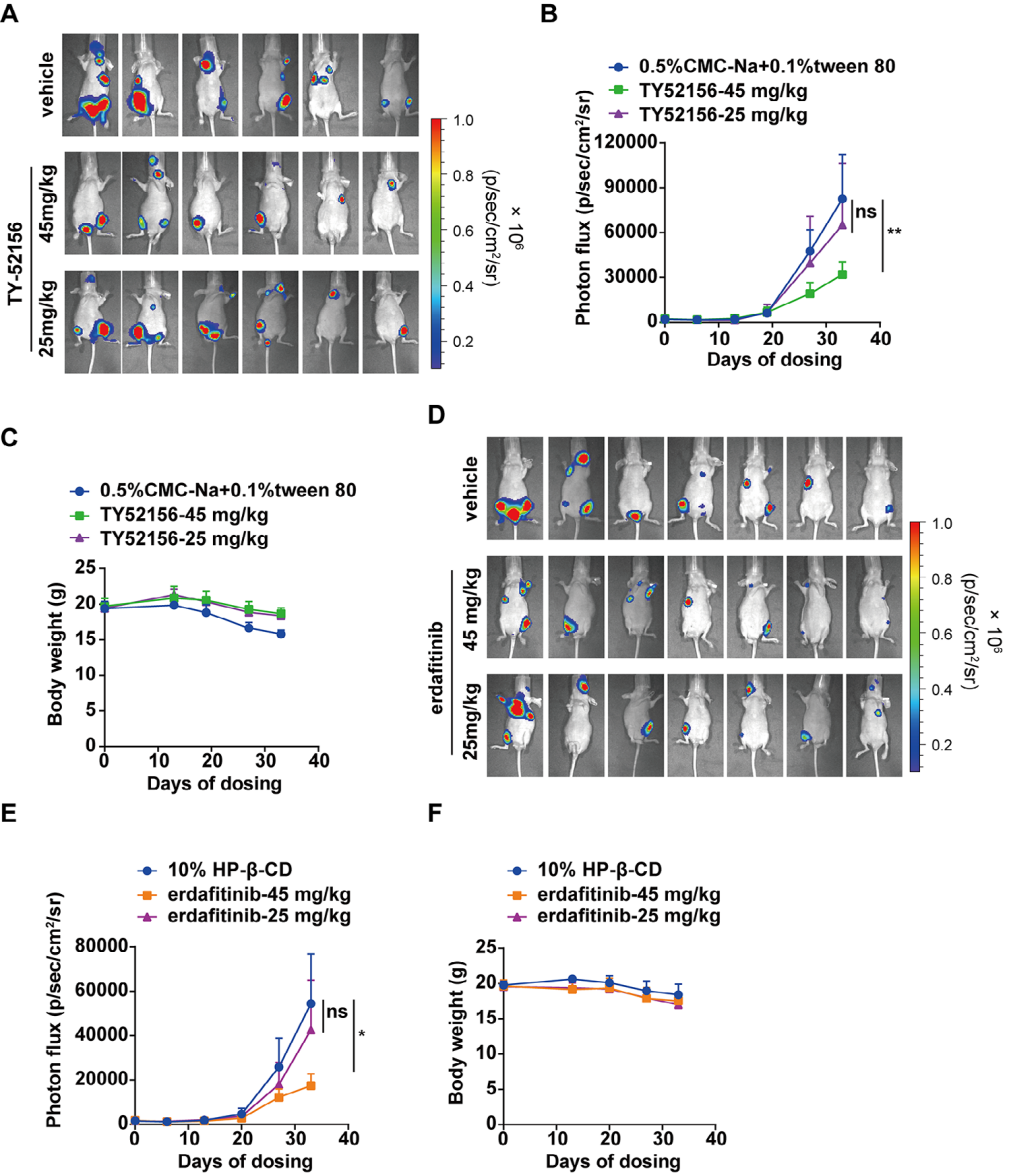
Pharmacologically targeting S1PR3 or FGFR1 prevents metastasis in animal models of lung cancer via intracardiac injection. (A) The in vivo metastasis model was established by intracardiac injection of luciferase‐labeled 120067R cells in immunodeficient BALB/c nude mice. Animals were orally administrated daily with vehicle or TY‐52156 (45 mg/kg and 25 mg/kg), and a bioluminescence signal was detected by the IVIS spectrum in vivo imaging system once a week. (B) Quantitation of bioluminescence imaging data in (A). (C) The body weight of mice in the TY‐52156‐treated group was measured once a week. (D) Animals were orally administrated daily with vehicle or erdafitinib (45 mg/kg and 25 mg/kg). (E) Quantitation of bioluminescence imaging data in (D). (F) Body weight of mice in erdafitinib‐treated group. The significance of quantitation of bioluminescence imaging data was measured using two‐way ANOVA. *p < 0.05, **p < 0.01, *** *p* < 0.001.

Given the involvement of the FGFR1‐S1PR3 axis in mediating metastasis in EGFR‐TKI‐resistant lung cancer cells, we test whether targeting S1PR3 or FGFR1 could prevent metastasis in vivo. We constructed 120067R cells to express luciferase stably and injected them into immunodeficient BALB/c nude mice via intracardiac injection. The mice were then randomized into the vehicle or S1PR3 inhibitor TY‐52156 (45 and 25 mg/kg) treatment groups and a long‐term treatment study was initiated. As expected, treatment with S1PR3 inhibitor TY‐52156 at 45 mg/kg effectively decreased metastasis compared to vehicle‐treated groups, whereas the 25 mg/kg dose showed a non‐significant reduction trend (Figure 6A). The quantitation of bioluminescent imaging signals confirmed the dose‐dependent suppression of metastatic outgrowth of mice bearing 120067R cells (Figure 6B). In addition, vehicle‐treated animals displayed slight body weight loss, indicating tumor progression, whereas the body weight remained stable in TY‐52156‐treated animals during the study (Figure 6C).

Since FGFR1 has been identified as an important regulatory protein of S1PR3 and several FGFR1 inhibitors have been marketed, we then explored whether targeting FGFR1 could effectively suppress resistant tumor metastasis in vivo. Bioluminescence imaging and quantitation results indicated that erdafitinib at high‐dose groups (45 mg/kg) effectively inhibited 120067R cell metastasis in vivo (Figure 6D,E) with no obvious body weight loss (Figure 6F). The in vivo data demonstrated that inhibition of S1PR3 or FGFR1 effectively suppressed metastasis, which may provide therapeutic strategies for patients with EGFR‐resistant lung cancer.

## 3 Discussion

Lung cancer is a highly malignant tumor characterized by aggressive metastasis. Although third‐generation EGFR inhibitors have improved treatment outcomes, patients inevitably develop metastasis after resistance [39]. Here, we provided the first evidence that S1PR3 served as a common driver of metastasis in third‐generation EGFR inhibitors‐resistant lung cancer. S1PR3 was markedly upregulated in resistant cells and tumor samples from EGFR TKI‐resistant patients, and its high expression correlated with poor overall survival. Functional studies demonstrated that S1PR3 promotes migration and invasion of resistant cells, whereas its depletion suppresses these phenotypes. Mechanistically, S1PR3 activates the RAC1‐PAK1 pathway to drive EMT progression, thereby facilitating metastatic potential.

The mechanisms underlying S1PR3 upregulation and its role in metastasis in EGFR TKI‐resistant cells remained unclear. Previous studies have implicated FGFR1 in tumor metastasis and EGFR TKI resistance, yet its relationship with S1PR3 has not been defined. Here, we observed a positive correlation between FGFR1 and S1PR3 expression in lung adenocarcinoma patients from TCGA datasets. Functional analyses showed that FGFR1 depletion led to decreased mRNA and protein expression of S1PR3, indicating that FGFR1 regulates S1PR3 expression at the transcriptional level. Bioinformatic prediction identified STAT4 as a potential transcription factor for S1PR3. Although STAT4 expression has been previously restricted to hematopoietic cells [40], its role in tumorigenesis and metastasis has not been extensively studied. Our data showed enhanced expression of STAT4 in resistant cells, and STAT4 inhibition suppressed cell migration, invasion, and S1PR3 expression, while FGFR1 inhibition reduced STAT4 levels. These findings indicate that FGFR1 regulates S1PR3 transcription through STAT4. In vivo inhibition of the FGFR1‐S1PR3 axis significantly suppressed metastasis. Although high‐dose erdafitinib or TY‐52156 monotherapy reduced metastatic burden, complete tumor regression was not achieved, suggesting that dual targeting of the FGFR1‐S1PR3 vertical pathway may provide improved therapeutic efficacy. This hypothesis currently under investigation in our future research. While our study provides new insights into the mechanisms of metastasis in EGFR TKI‐resistant lung cancer, the precise role of S1PR3 in mediating resistance warrants further investigation. Besides, the potential role of S1PR3 in regulating metastasis in other types of tumors presents an exciting avenue for future research.

**FIGURE 7:**
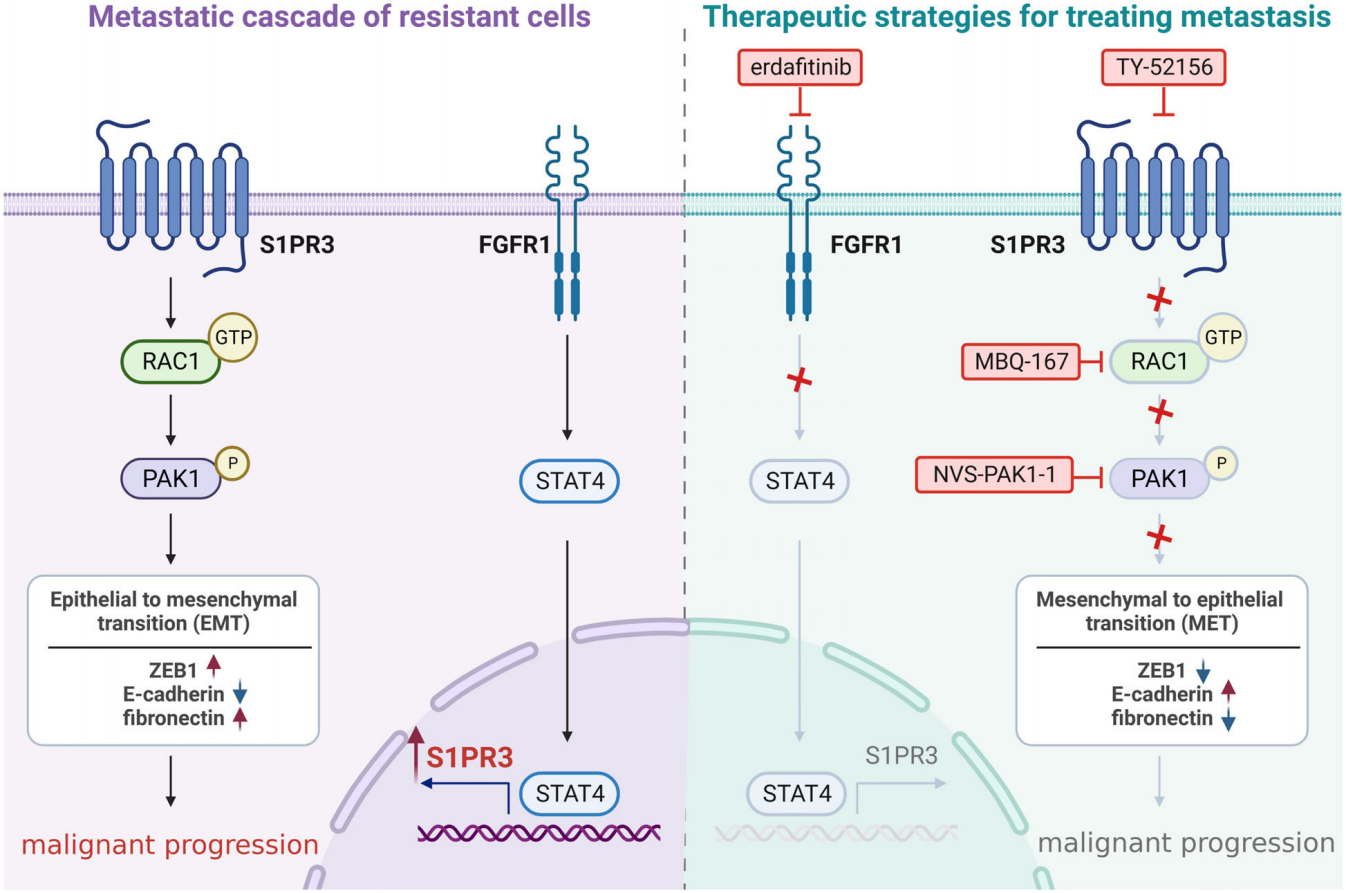
Mechanistic schematic of the FGFR1‐STAT4‐S1PR3‐RAC1‐PAK1‐EMT axis in EGFR TKI‐resistant models. The figure was created with BioRender.com.

In summary, our study elucidated a novel FGFR1‐STAT4‐S1PR3‐RAC1‐PAK1‐EMT signaling axis in the metastasis of EGFR TKI‐resistant lung cancer cells, which has not been previously reported (Figure 7). Our findings may provide valuable insights into therapeutic strategies for managing metastatic recurrence in EGFR TKI‐treated patients.

## 4 Materials and Methods

### 4.1 Cell Culture and Compounds

NCI‐H1975 cells were obtained from the American Type Culture Collection (ATCC). The 120067‐resistant 120067R or AZD9291‐resistant AZD9291R cells were derived from NCI‐H1975 cells, as described previously [8]. All cell lines were cultured in RPMI‐1640 medium (Gibco) with 10% FBS (Gibco) at 37°C in 5% CO_2_, and short tandem repeat (STR) analysis was performed by Genesky.

Compound ASK120067 is a novel and selective third‐generation EGFR TKI developed in our laboratory. AZD9291 was purchased from Selleck Chemicals (#S7297).

### 4.2 RNA‐seq Analysis

Three independent samples were collected and submitted for sequencing by Shanghai Majorbio Bio‐Pharm Technology, China. GO enrichment analysis of RNA‐seq data was performed by DAVID (https://david.ncifcrf.gov/).

### 4.3 Trans‐well Migration and Invasion Assay

Trans‐well assay was performed in 24‐well trans‐well insets with 8.0‐µm pore size. For the migration assay, 3 × 10^4^ cells were suspended in 100 µL of serum‐free RPMI‐1640 medium and plated onto the top of the filter membrane. Then 600 µL RPMI‐1640 medium with 10% FBS was added into the bottom of the lower chamber. For the invasion assay, matrigel (100 µL) was added to the trans‐well insert and solidified in a 37°C incubator for 2 h to form a thin gel layer. Then, an excess of matrigel was removed, and 6 × 10^4^ cells with serum‐free medium were seeded into the inner chamber. After incubation for 12 h, cells were fixed with 90% ethanol for 15 min and then stained with 0.1% crystal violet for another 15 min. Subsequently, the crystal violet was gently removed from the top of the membrane with a cotton swab and captured with a microscope at 20×. For the quantification, the migration or invasion cells were dissolved with 30% glacial acetic acid and measured by SoftMax Pro software at the absorbance of 570 nm.

### 4.4 RNA Isolation and RT‐PCR Assay

Total RNA was extracted from cultured cells using an EZ‐press RNA Purification Kit (EZBioscience, #B0004D) according to the manufacturer's instructions. Then RNA was reverse‐transcribed to cDNA using HiScript II Q RT SuperMix for qPCR kit (Vazyme, #R223‐01). RT‐PCR assay was performed with ChamQ Universal SYBR qPCR Master Mix (Vazyme, #Q711‐02). Data was analyzed by normalization against actin. The primer used are listed in Table S3.

### 4.5 Immunoblotting

Immunoblotting was performed as described previously [41]. Cells were collected and lysed in SDS lysis buffer. Tumor tissue was homogenized in RIPA buffer with a phosphatase inhibitor cocktail (Roche, #4906845001) and protease inhibitor cocktail (Roche, #4693132001) on ice. After determining the protein concentration using the BCA protein assay (Thermo Fisher Scientific, #23227), protein samples were heated at 100°C for 15 min and separated on SDS‐PAGE gels. Then the protein band was transferred to nitrocellulose membranes and immunoblotted with primary antibodies against S1PR3 (abcam, #ab108370), RAC1 (PTM BIO, #5329), p‐PAK1 (CST, #2601S), PAK1 (CST, #2602S), ZEB1 (CST, #70512S), E‐cadherin (CST, #3195S), fibronectin (CST, #26836S), FGFR1 (CST, #9740S), STAT4 (CST, #2653S), and actin (Proteintech, 60008‐1‐Ig) at 4°C overnight and then incubated with secondary antibodies (Jackson, 111‐035‐003).

### 4.6 siRNA‐Mediated Knockdown Assay

The small interfering RNA (siRNA) targeting human genes (S1PR3, FGFR1, STAT4) and negative control (NC) were synthesized by Obio Technology (Shanghai, China). The sequences used are listed in Table S4.

Seeded cells to be 60%–80% confluent at the time of transfection in a six‐well plate. Lipofectamine RNAiMAX reagent and siRNA were diluted in opti‐MEM medium, respectively. After incubating for 5 min at room temperature, lipofectamine RNAiMAX was added to each well containing the diluted RNAi molecules and incubated for 10–20 min. Then, siRNA‐lipofectamine RNAimax complex was added to cells for 24 h. Subsequently, the transfected cells could be analyzed.

### 4.7 H&E Staining and IHC

Fixed clinical samples were obtained from Shanghai Chest Hospital, Shanghai Jiao Tong University, and Shanghai Institute of Biochemistry and Cell Biology, Chinese Academy of Sciences. Tumor tissues from mice were collected and treated with 4% paraformaldehyde. H&E staining and IHC were performed by Shanghai ZuoChengBio, China.

### 4.8 Chromatin Immunoprecipitation (ChIP) Assay

ChIP assay was performed using the ChIP assay kit (CST, #9005) following the manufacturer's instructions. Antibodies used were as follows: Normal Rabbit IgG (CST, #2729S) and STAT4 (CST, #2653S). The S1PR3‐specific primers used are listed in Table S5.

### 4.9 Promoter Luciferase Reporter Assay

HEK293T cells were transfected with STAT4 and S1PR3 luciferase reporter plasmid using Lipofectamine 3000 Transfection Reagent (Thermo Fisher Scientific, #18324012) for 24 h. Luciferase activity was performed using a Dual‐Luciferase Reporter Gene Assay Kit (Yeasen Biotechnology, #11402ES60). Luciferase activity was calculated by normalizing to Renilla luciferase activity.

### 4.10 In Vivo Luciferase Imaging Assay

To analyze the effect of S1PR3 on long‐term metastasis, female BALB/c nude mice have intracardiac injected with 1 × 10^6^ 120067R cells. Animals were monitored for any signs of distress following the injection of tumor cells and then randomly assigned to vehicle or treatment groups. TY‐52156 was reconstituted in 0.5% methylcellulose with 0.1% tween 80, and erdafitinib was reconstituted in 10% HP‐β‐CD at the indicated doses. All animals were administrated once a day by oral gavage. An in vivo imaging system (IVIS, PerkinElmer) was used to monitor metastasis weekly. Animal studies were approved by the Institutional Animal Care and Use Committee of the Shanghai Institute of Materia Medica.

### 4.11 Bioinformatic Analysis

S1PR3 expression data were obtained from The Cancer Genome Atlas (TCGA) Research Network through cBioportal dataset ((http://cbioportal.org).

### 4.12 Statistical Analysis

Data were analyzed using GraphPad Prism 8.0 software. Quantitative data were analyzed by a two‐tailed Student's *t*‐test or two‐way ANOVA. Statistical significance was defined as *p < 0.05, **p < 0.01, and ***p < 0.001.

## Author Contributions

Mengzhen Lai, Jiaying Chen, and Zilu Pan performed the experiments, collected the data, and analyzed it. Mengzhen Lai prepared the manuscript. Ye Qin provided the intracardiac injection techniques in animal experiments. Hui Zhang provided a clinical sample and analyzed the data. Tao Zhang constructed resistant cells and revised the manuscript. Linjiang Tong, Yan Li, and Fang Feng performed animal administration and IVIS detection. Haotian Tang, Gang Bai, Peiran Song, Yingqiang Liu, Yi Chen, and Yan Fang provided technical support. Qiupei Liu, Ye Qin, Meiyu Geng, Hao Chen, Bencan Tang, and Ker Yu reviewed the manuscript. Hua Xie, Jian Ding, and Ker Yu designed the study and reviewed the manuscript. All authors have read and approved the final manuscript.

## Funding

This work was supported by grants from the Special Funds of the National Natural Science Foundation of China (82441046), National Natural Science Foundation of China (82273948, 82071976), the Project of Shanghai Institute of Materia Medica, Chinese Academy of Sciences (No. SIMM0120231001), Opening Foundation SKLDR‐2023‐TT‐01 and SIMM2205KF‐09 from State Key Laboratory of Drug Research, National Key Research and Development Program of China (2024YFA1210200), High‐level new R&D Institute (2019B090904008), High‐level Innovative Research Institute (2021B0909050003), and Department of Science and Technology of Guangdong Province, Zhongshan Municipal Bureau of Science and Technology (2023B2029).

## Ethics Statement

All animal studies were approved by the Institutional Animal Care and Use Committee (IACUC: 2023‐01‐DJ‐73) and strictly performed according to the institutional ethical guidelines on animal care.

## Conflicts of Interest

The authors declare no conflicts of interest.

## Supporting information




**Supporting File 1**: mco270744‐sup‐0001‐SupMat.docx.

## Data Availability

All data are available from the corresponding authors upon request.
