# Supplementary material for: Sphingosine‐1‐Phosphate Receptor 3 Confers Tumor Metastasis in Lung Cancer Resistant to Third‐Generation EGFR Inhibitor
Source: MedComm (2020). 2026 Apr 23;7(5):e70744. doi: 10.1002/mco2.70744 (PMC13106892; doi:10.1002/mco2.70744)
Supplement: Supplementary file 1 — Supporting File 1: mco270744‐sup‐0001‐SupMat.docx. [file MCO2-7-e70744-s001.docx]

**Supporting Information**

**Sphingosine-1-phosphate receptor 3 confers tumor metastasis in lung cancer resistant to third-generation EGFR inhibitor**

**Running title: S1PR3 drives metastasis in resistant lung cancer**

Mengzhen Lai^a,b,e,1^, Jiaying Chen^a,^^e,1^, Ye Qin^d,1^, Hui Zhang^f,1^, Zilu Pan^a,e^, Tao Zhang^a^, Linjiang Tong^a^, Haotian Tang^a,c^, Gang Bai^a^, Qiupei Liu^a,g^, Yan Li^a^, Fang Feng^a^, Peiran Song^a^, Yingqiang Liu^a^, Yi Chen^a,e^, Yan Fang^a,e^, Bencan Tang^g^, Meiyu Geng^a,e^, Ker Yu^b*^, Hao Chen^d*^, Jian Ding^a,b,e*^ and Hua Xie^a,c,e*^

*^a^*Division of Antitumor Pharmacology & State Key Laboratory of Drug Research, Shanghai Institute of Materia Medica, Chinese Academy of Sciences, Shanghai 201203, China.

*^b^*Department of Pharmacology, School of Pharmacy, Fudan University, Shanghai 201203, China.

*^c^*Zhongshan Institute for Drug Discovery, Shanghai Institute of Materia Medica, Chinese Academy of Sciences, Zhongshan 528400, China.

*^d^*State Key Laboratory of Chemical Biology, Molecular Imaging Center, Shanghai Institute of Materia Medica, Chinese Academy of Sciences, Shanghai 201203, China.

*^e^*University of Chinese Academy of Sciences, Beijing 100049, China.

*^f^*Shanghai Lung Cancer Center, Shanghai Chest Hospital, Shanghai Jiao Tong University School of Medicine, Shanghai 200030, China.

*^g^*Key Laboratory for Carbonaceous Waste Processing and Process Intensification Research of Zhejiang Province, Department of Chemical and Environmental Engineering, The University of Nottingham Ningbo China, Ningbo 315100, China.

^1^These authors contributed equally to this work.

^*^Corresponding authors: Hua Xie, E-mail: [hxie@simm.ac.cn](mailto:hxie@simm.ac.cn); Jian Ding, E-mail: [jding@simm.ac.cn](mailto:jding@simm.ac.cn); Hao Chen, E-mail: [haoc@simm.ac.cn](mailto:haoc@simm.ac.cn); Ker Yu, E-mail: [keryu@fudan.edu.cn](mailto:keryu@fudan.edu.cn).


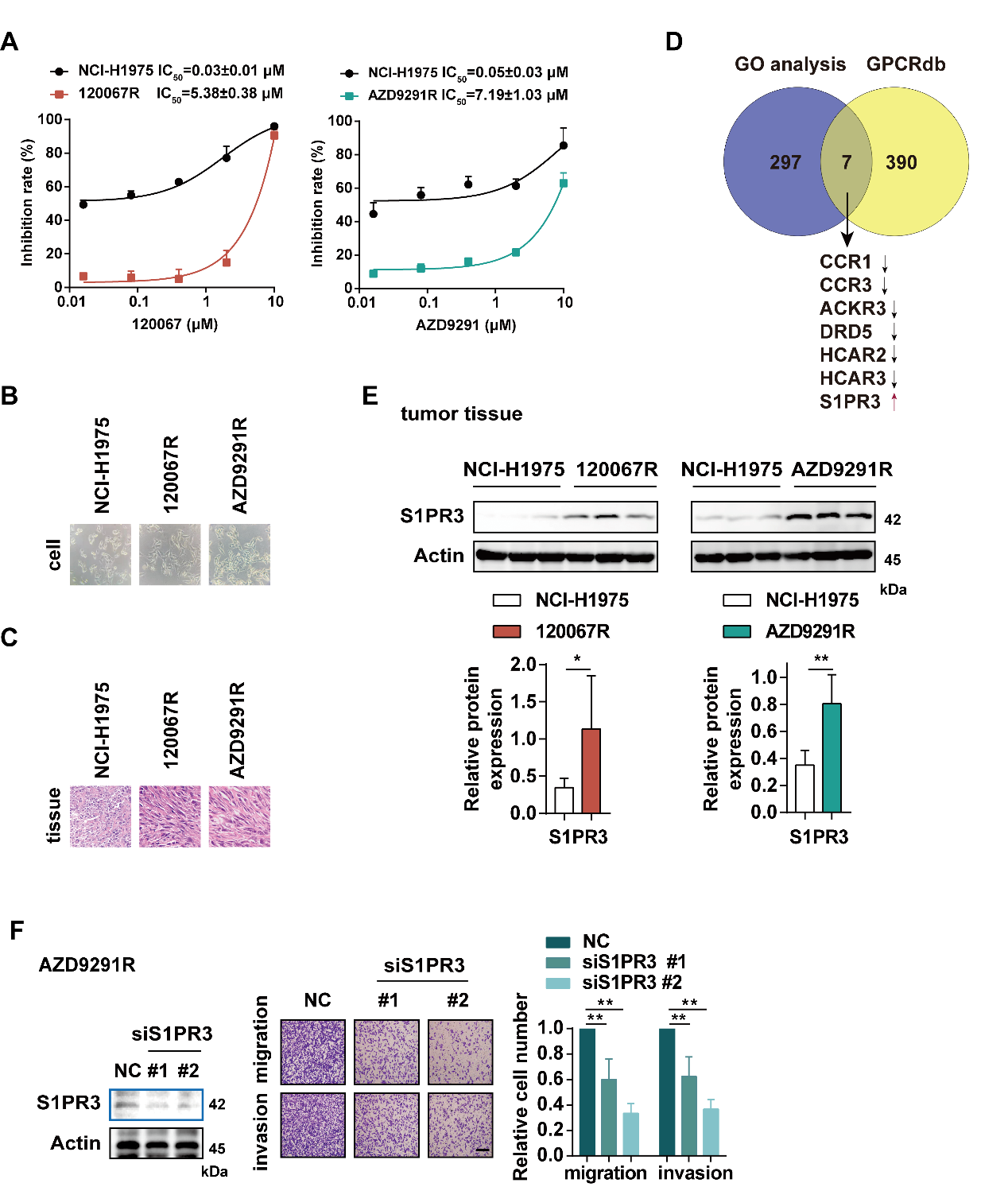


**FIGURE S1** S1PR3 is a key mediator of metastasis in EGFR inhibitor-resistant lung cancer cells. (A) Drug resistance evaluation in 120067R and AZD9291R resistant cell lines. (B) The cell morphology of parental and resistant cells. (C) The cell morphology of parental and resistant cells xenograft tumor tissues by H&E staining. (D) The Venn diagram of GO-derived metastasis-related gene signatures and GPCR genes from GPCRdb. (E) Protein level of S1PR3 in NCI-H1975 xenograft tumor tissues, 120067R xenograft tumor tissues and AZD9291R xenograft tumor tissues. (F) Migration and invasion of AZD9291R cells after siS1PR3 transfection. Scale bar, 100 µm. ** *p* < 0.01.


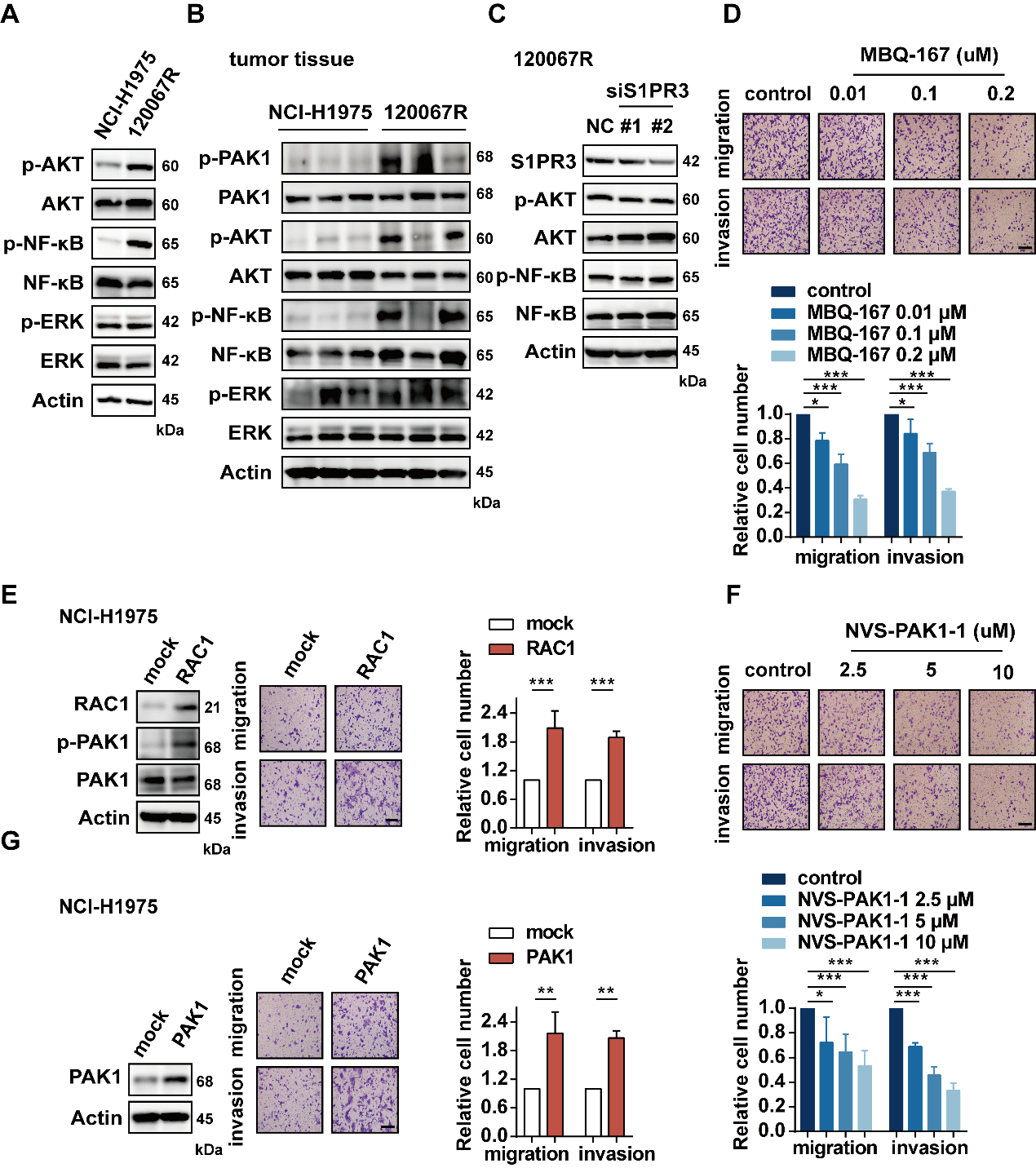
 **FIGURE S2** S1PR3 contributed to metastasis in EGFR resistant lung cancer cells through RAC1-PAK1 signaling pathway. (A-B) The protein expression of NCI-H1975 cells, 120067R cells (A) or NCI-H1975 and 120067R xenograft tumor tissues detected by immunoblotting (B). (**C**) Immunoblotting on 120067R cells after S1PR3 knockdown. (D) Migration and invasion of 120067R cells treatment with RAC1 inhibitor MBQ-167. (E) Migration and invasion of NCI-H1975 cells exogenous overexpressed RAC1. (F) Migration and invasion of 120067R cells treatment with PAK1 inhibitor NVS-PAK1-1. (G) Migration and invasion of NCI-H1975 cells exogenous overexpressed PAK1. Scale bar, 100 µm. **p* < 0.05, ***p* < 0.01, *** *p* < 0.001.


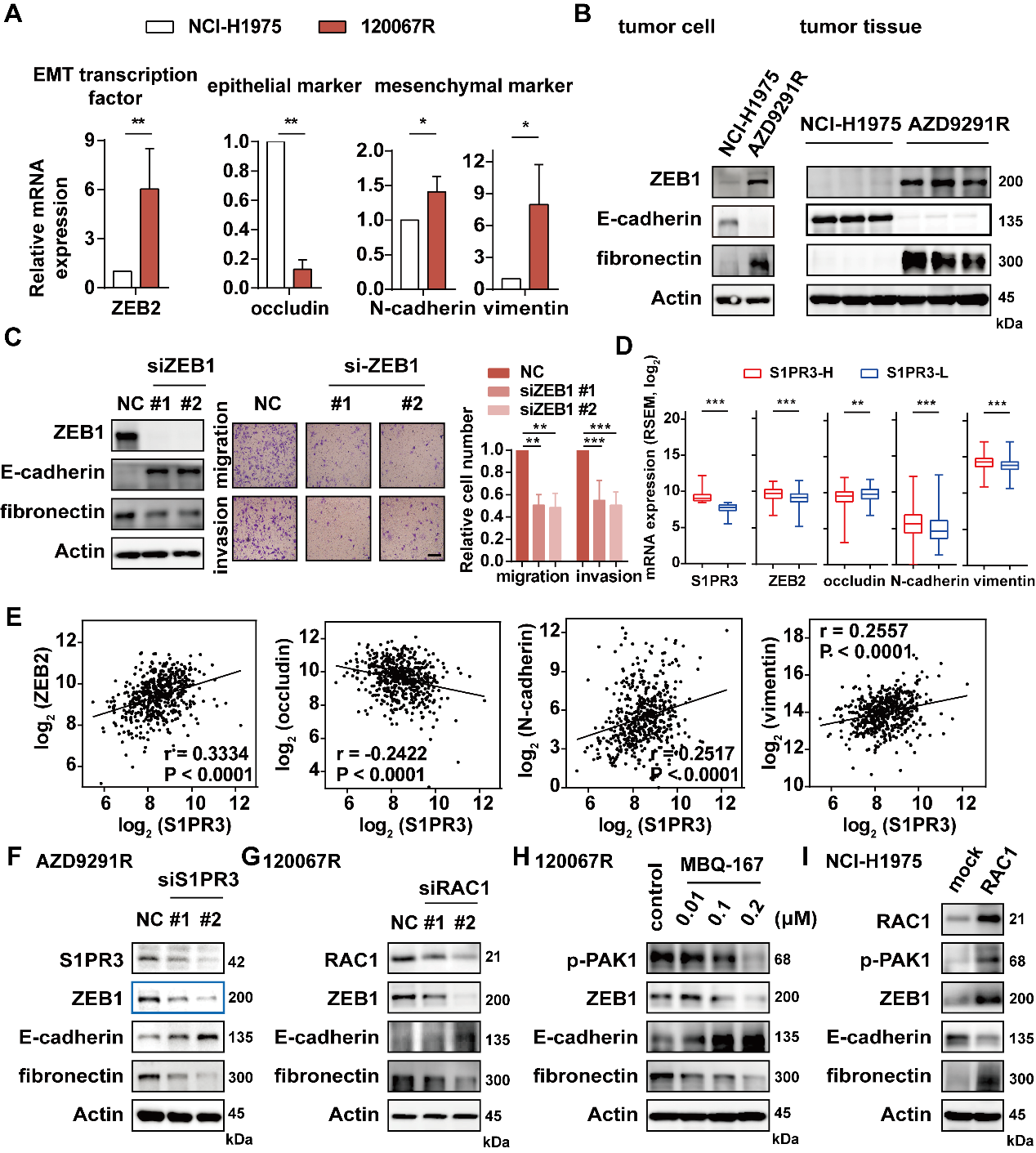
 **FIGURE S3** S1PR3-RAC1-PAK1 axis is associated with EMT progression. (A-B) Gene (A) or protein (B) expression of EMT-related markers. (C) Migration and invasion of 120067R cells after ZEB1 knockdown. (D) Relative mRNA level of EMT-related genes in S1PR3^high^ group (S1PR3-H) and S1PR3^low^ group (S1PR3-L) of lung adenocarcinoma patients from TCGA database. (E) The association between S1PR3 and EMT-related genes level in lung adenocarcinoma patients from TCGA database. (F) Immunoblotting on AZD9291R cells after S1PR3 knockdown. (G-H) Immunoblotting on 120067R cells after RAC1 knockdown (G) or treatment with RAC1 inhibitor MBQ-167 (H). (I) Immunoblotting on NCI-H1975 cells exogenous overexpressed RAC1. Scale bar, 100 µm. **p* < 0.05, ***p* < 0.01, *** *p* < 0.001.


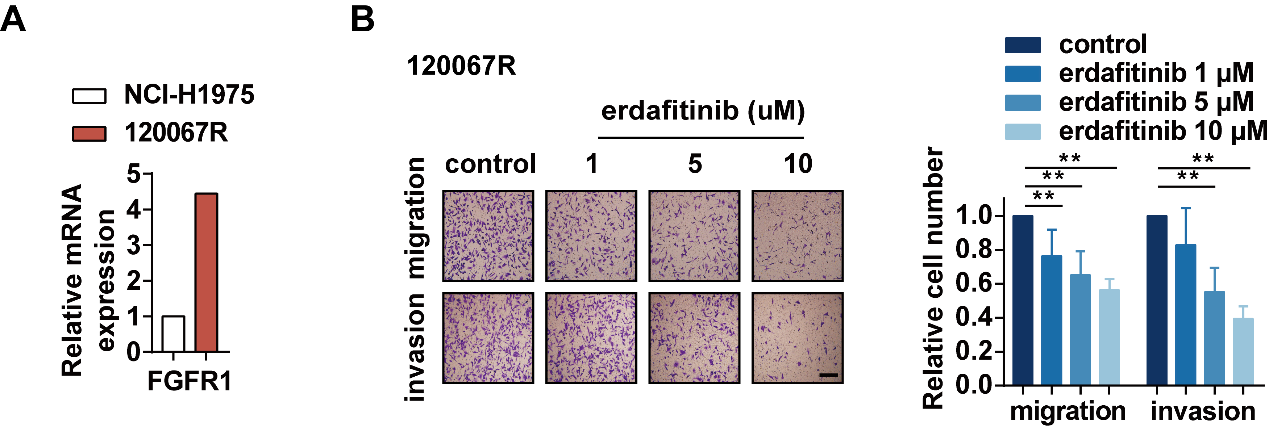
 **FIGURE S4** FGFR1 mediated 120067R cells metastasis. (A) Relative mRNA expression of FGFR1 in NCI-H1975 cells and 120067R cells by RNA-seq analysis. (B) Migration and invasion of 120067R cells treated with FGFR1 inhibitor erdafitinib. Scale bar, 100 µm. ***p* < 0.01.


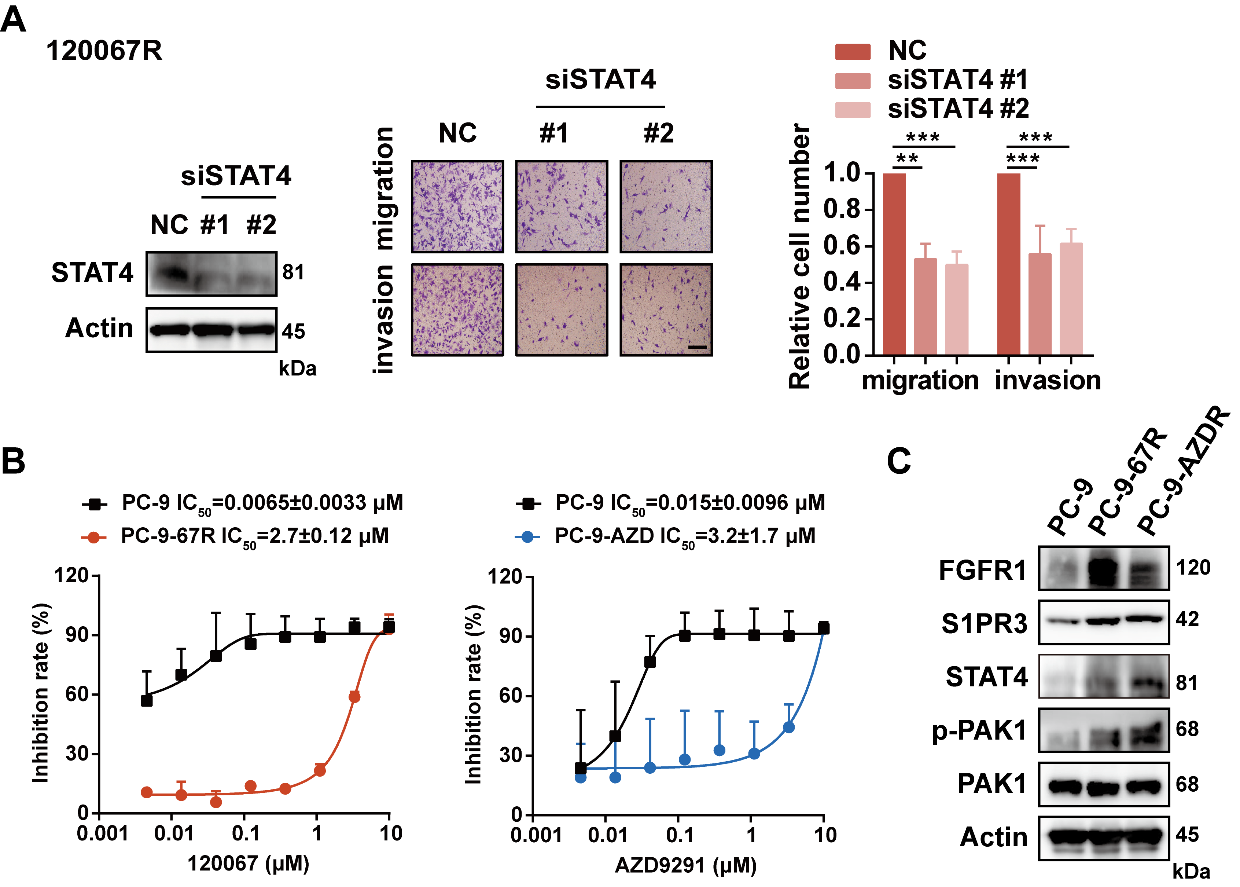


**FIGURE S5** Generalizability of the FGFR1-STAT4-S1PR3-PAK1 signaling axis across an additional third-generation EGFR-TKI-resistant cell model. (A) Migration and invasion of 120067R cells after STAT4 knockdown. (B) Drug resistance evaluation in PC-9 cells-derived 120067- and AZD9291-resistant cell lines. (C) Protein level of FGFR1, S1PR3, STAT4, p-PAK1 and PAK1 in parental PC-9 cells and resistance cells.

**TABLE S1** Tumor metastasis-related signatures from GO enrichment and GPCR genes from GPCRdb.

| GO analysis | SEMA5A, RIPOR2, COL16A1, COL12A1, ICAM5, F11R, CLDN1, ICAM1, LOXL2, CDH3, CDH2, BSG, CCN5, CYP1B1, SVEP1, CCN2, EPHB4, SCN1B, CD177, PCDHAC1, MAGI1, EPHA4, PCDHA10, AZGP1, CLDN4, CLDN9, CLDN7, LY6D, COL8A1, TLN2, DSC2, FBLN7, CD99L2, VTN, EFNB1, ADGRG1, PODXL, FLRT1, SLAMF7, PCDHA3, IGFBP7, MYH10, PCDHA6, COL28A1, CCR1, MUC16, JUP, LRRN2, MCAM, FN1, PARVA, PARVB, L1CAM, COL1A1, SELL, COL5A1, PCDHB2, ITGA11, FAT1, ITGBL1, PCDHB3, PCDHB9, CD22, FERMT2, DDR1, CNTNAP3, ITGB1, ACHE, CNTNAP1, ITGAM, ITGB4, TGFB1I1, PCDHGB2, ITGB2, HAPLN3, ROBO1, ADAMTSL1, ADGRE2, ADGRE5, ITGAX, EMILIN2, ITGB8, ITGB7, DGCR6, ITGB6, NEO1, CCR3, PCDHGA8, EGFL7, ITGA3, ITGA2, EMP2, RGMB, PARD3B, RHOB, CLDN11, CLDN10, CEACAM1, COL6A1, PXDN, MMRN2, CDH11, ITGA6, TNXB, LAMA3, NEDD9, THBS3, PRTG, GPNMB, CCL2, PCDH1, IL32, PCDHGC5, PCDHB13, LAMB1, GRHL2, SORBS3, THEMIS2, CD9, ACKR3, NECTIN4, NECTIN3, LAMA5, TENM2, TTYH1, CELSR1, VSIG10, LRP6, TMEM47, SRPX2, BAIAP2L1, CLEC7A, TRIM29, CDH1, EPCAM, MPZL2, KIRREL1, ITGA1, VNN1, DLG3, DLG4, CDHR2, PKP2, BCL2, FAT2, FAT4, PKP3, TJP3, COL17A1, EDA, ECM2, LYPD3, NID1, TNN, MUC4, SORBS1, FREM1, SFTA3, SCARB1, CDKN1A, IL24, ALOX15, TGFA, FGF1, EVPL, PPL, TSKU, ERBB3, SMPD1, KRT6A, DRD5, PDGFRB, SERPINB2, TPM1, NOG, WNT5A, ARHGEF19, GRHL3, EREG, DCBLD2, LOX, PATJ, DOC2A, AMOT, CPNE3, CXADR, SLC2A11, MAP1S, ACTN1, PLEKHG6, TBC1D2, SYNPO2L, IRF6, LCP1, ARHGEF5, SLC7A2, HCAR2, HCAR3, SCIN, MARVELD2, EPB41L2, EPB41L3, PAK6, TSPAN1, SPTBN2, MFAP3L, TCAF2, FYB1, KDF1, SAMD4A, CGN, MPP7, TSPAN15, OCLN, GJB3, CRB3, FOXA2, TP73, CLIC4, PTPRJ, CD53, PRKCG, PRKCH, HEG1, PRKCD, CNKSR1, PRKD1, STEAP1, HPN, TWF1, ADD3, CORO1A, SV2A, MPP1, CADM1, MAPK15, P2RX7, VANGL2, DES, PTPN6, NECTIN2, CIB2, SYK, ADAM11, HSPG2, MMP14, FRMD5, IL1B, SEMA7A, PTN, S1PR3, GFAP, ARPC1B, ARPC1A, WIPF3, DIXDC1, FMN1, MYLK, SYNE1, CSRP3, PSTPIP2, DAG1, TNS4, TNS1, TPM4, LIMCH1, SHROOM2, MYO7A, INF2, MYO3B, MAP1B, MAP1A, S100A4, PFN2, TAGLN, PLEKHH2, CNN1, ABLIM2, PDLIM2, CLMN, EPB41L1, MYO6, TNNI1, EPS8L1, TNNI3, CAP2, ACE, GSN, KLHL2, MICAL2, MSN, KLHL3, KLHL4, FHDC1, SSH3, MYO1D, SMTN, MLPH, MYO15B, HOOK1, MYO5B, TRPV4, JMY, MYO5C |
| --- | --- |
| GPCRs | CNR1, CNR2, FFAR1, FFAR2, FFAR3, FFAR4, GPR42, GPR18, GPR55, GP119, LT4R1, LT4R2, CLTR1, CLTR2, OXER1, LPAR1, LPAR2, LPAR3, LPAR4, LPAR5, LPAR6, S1PR1, S1PR2, S1PR3, S1PR4, S1CCR1PR5, PTAFR, PD2R, PD2R2, PE2R1, PE2R2, PE2R3, PE2R4, PF2R, PI2R, TA2R, MTR1A, MTR1B, AA1R, AA2AR, AA2BR, AA3R, P2RY1, P2RY2, P2RY4, P2RY6, P2Y11, P2Y12, P2Y13, P2Y14, US28, AGTR1, AGTR2, APJ, MMBR, GRPR, BRS3, BKRB1, BKRB2, CCKAR, GASR, C3AR, C5AR1, C5AR2, EDNRA, EDNRB, FPR1, FPR2, FPR3, GALR1, GALR2, GALR3, GHSR, GNRHR, KISSR, MCHR1, MCHR2, MSHR, ACTHR, MC3R, MC4R, MC5R, MTLR, NMUR1, NMUR2, NPFF1, NPFF2, NPSR1, NPBW1, NPBW2, NPY1R, NPY2R, NPY4R, NPY5R, NPY6R, NTR1, NTR2, OPRD, OPRK, OPRM, OPRX, OX1R, OX2R, QRFPR, PRLHR, PAR1, PAR2, PAR3, PAR4, RXFP1, RXFP2, RL3R1, RL3R2, SSR1, SSR2, SSR3, SSR4, SSR5, NK1R, NK2R, NK3R, TRFR, UR2R, V1AR, V1BR, V2R, OXYR, CML1, CML2, CCR1, CCR2, CCR3, CCR4, CCR5, CCR6, CCR7, CCR8, CCR9, CCR10, CXCR1, CXCR2, CXCR3, CXCR4, CXCR5, CXCR6, CX3C1, XCR1, ACKR1, ACKR2, ACKR3, ACKR4, CCRL2, FSHR, LSHR, TSHR, PKR1, PKR2, OPSD, OPN3, OPN4, OPN5, OPSB, OPSG, OPSR, OPSX, GPBAR, GPER1, HCAR1, HCAR2, HCAR3, OXGR1, SUCR1, 5HT1A, 5HT1B, 5HT1D, 5HT1E, 5HT1F, 5HT2A, 5HT2B, 5HT2C, 5HT4R, 5HT5A, 5HT5B, 5HT6R, 5HT7R, ACM1, ACM2, ACM3, ACM4, ACM5, ADA1A, ADA1B, ADA1D, ADA2A, ADA2B, ADA2C, ADRB1, ADRB2, ADRB3, DRD1, DRD2, DRD3, DRD4, DRD5, HRH1, HRH2, HRH3, HRH4, TAAR1, GPR3, GPR4, GPR6, GPR12, GPR15, GPR17, GPR19, GPR20, GPR21, GPR22, GPR25, GPR26, GPR27, GPR31, GPR32, GPR33, GPR34, GPR35, GPR37, G37L1, GPR39, GPR45, MTR1L, GPR52, GPR61, GPR62, GPR63, PSYR, OGR1, GPR75, GPR78, GPR82, GPR83, GPR84, GPR85, GPR87, GPR88, GP101, GP132, GP135, GP139, GP141, GP142, GP146, GP148, GP149, GP150, GP151, GP152, GP153, GP160, GP161, GP162, GP171, GP173, GP174, GP176, GP182, GP183, LGR4, LGR5, LGR6, MAS, MAS1L, MRGRD, MRGRE, MRGRF, MRGRG, MRGX1, MRGX2, MRGX3, MRGX4, P2RY8, P2Y10, TAAR2, TAAR3, TAAR5, TAAR6, TAA7F, TAAR8, TAAR9, TAAR4, CRFR1, CRFR2, GHRHR, GIPR, GLP1R, GLP2R, GLR, SCTR, PTH1R, PTH2R, PACR, VIPR1, VIPR2, CALCR, CALRL, AGRA1, AGRA2, AGRA3, AGRB1, AGRB2, AGRB3, CELR1, CELR2, CELR3, AGRD1, AGRD2, AGRE1, AGRE2, AGRE3, AGRE4, AGRE5, AGRF1, AGRF2, AGRF3, AGRF4, AGRF5, AGRG1, AGRG2, AGRG3, AGRG4, AGRG5, AGRG6, AGRG7, AGRL1, AGRL2, AGRL3, AGRL4, AGRV1, FZD1, FZD2, FZD3, FZD4, FZD5, FZD6, FZD7, FZD8, FZD9, FZD10, OWS, TA2R1, TA2R3, TA2R4, TA2R5, TA2R7, TA2R8, TA2R9, T2R10, T2R13, T2R14, T2R16, T2R19, T2R20, T2R30, T2R31, T2R38, T2R39, T2R40, T2R41, T2R42, T2R43, T2R45, T2R46, T2R50, T2R60, GABR1, GABR2, GRM1, GRM2, GRM3, GRM4, GRM5, GRM6, GRM7, GRM8, CASR, GP156, GP158, GP179, RAI3, GPC5B, GPC5C, GPC5D, GPC6A, TS1R1, TS1R2, TS1R3 |

**TABLE S2** Transcription factors upstream of S1PR3 predicted by JASPAR database and ALGGEN PROMO database

| JASPAR database | Nfat5, KLF4, STAT4, NFATC4, REL, Nfatc1, Zfp335, NR1D2, ZNF708, SOX1, OSR1, RELA, KLF5, Nfatc2, MZF1, PBX3, NR1D1, ZNF281, ZNF257, DUXA, TBX20, ERF::FOXI1, NFATC3, INSM1, Dux, RARA::RXRG, PHOX2A, KLF9, ZNF528, Prdm15, ZNF384, Prdm14, KLF16, SOX21, TWIST1, Spz1, ZNF454, PRDM9, Erg, Ikzf3, TFAP2A, EOMES, FLI1::FOXI1, HAND2, PATZ1, ZIC2, ZNF549, TBX18, ZNF410, MGA::EVX1, GABPA, Plagl1, STAT2 |
| --- | --- |
| ALGGEN PROMO database | C/EBPdelta, Msx-1, ZIC2, p300, FACB, MafG, Ncx, C/EBPalpha, STAT4, C/EBP, NF-1, DBP, HOXA3, En-1, Cdx-1, Pax-6, c-Ets-1, Hb, AGL3, YY1, STAT5A, TGGCA-binding protein, LIM1, S8, Cart-1, ZIC3, ZF5, LVc, VDR, ZIC1, MYB2, Elk-1, WT1 I, MZF-1, USF2, c-Ets-1 54, Nkx2-1, JunB, POU1F1a, Antp, Cutl1, R2, muEBP-C2, TFIIB, f(alpha)-f(epsilon), MYBAS1, MF3, AP-2alphaA, CREMtau, CREMtau1, CREMtau2, DREB1A, Pax-9a, Pax-9b, unc-86, NF-1 (-like proteins), C/EBPbeta, Pax-2a, FOXP3, Ovo-B, PBF, deltaCREB, DEF:GLO:SQUA, HNF-3beta, HNF-3alpha, Sox2, Tll, BR-C Z2, HELIOS, Crx, E2, Myf-3, MyoD, RC2, GA-BF, Pax-4a, Eve, HNF-3, MNB1a, PEA3, GAGA factor, GATA-2, Adf-1, ABI4, Nrf2:MafK, c-Jun, Pax-8, USF-1, LVb-binding factor, TMF, ABF1, PU.1, Zeste, CAC-binding protein, p53, DSXF, DSXM, HNF-1B, HNF-1C, TRM1, NF-AT1, E47, c-Ets-2, LCR-F1 |

**TABLE S3** Sequences of primers used for RT-PCR

| Genes | Primers |
| --- | --- |
| S1PR3 | F: 5-GTGATCCTCTACGCACGCATC-3 |
|  | R: 5-CGCTCCGAGTTGTTGTGGT-3 |
| ZEB1 | F: 5-GGCATACACCTACTCAACTACGG-3 |
|  | R: 5-TGGGCGGTGTAGAATCAGAGTC-3 |
| E-cadherin | F: 5-GCCTCCTGAAAAGAGAGTGGAAG-3 |
|  | R: 5-TGGCAGTGTCTCTCCAAATCCG-3 |
| fibronectin | F: 5-CCGCCGAATGTAGGACAAGA-3 |
|  | R: 5-TGCCTCTGCTGGTCTTTCAG-3 |
| ZEB2 | F: 5-CCTCTGTAGATGGTCCAGTGAA-3 |
|  | R: 5-GTCACTGCGCTGAAGGTACT-3 |
| occludin | F: 5-ATGGCAAAGTGAATGACAAGCGG-3 |
|  | R: 5-CTGTAACGAGGCTGCCTGAAGT-3 |
| N-cadherin | F: 5-CCTCCAGAGTTTACTGCCATGAC-3 |
|  | R: 5-GTAGGATCTCCGCCACTGATTC-3 |
| vimentin | F: 5-AGGCAAAGCAGGAGTCCACTGA-3 |
|  | R: 5-ATCTGGCGTTCCAGGGACTCAT-3 |
| FGFR1 | F: 5- TAATGGACTCTGTGGTGCCCTC-3 |
|  | R: 5- ATGTGTGGTTGATGCTGCCG-3 |
| STAT4 | F: 5- CAGTGAAAGCCATCTCGGAGGA-3 |
|  | R: 5- TGTAGTCTCGCAGGATGTCAGC-3 |
| Actin | F: 5-CACCATTGGCAATGAGCGGTTC-3 |
|  | R: 5-AGGTCTTTGCGGATGTCCACGT-3 |

**TABLE S4** siRNA sequences targeting human S1PR3, FGFR1, and STAT4

| Genes | Sequences |
| --- | --- |
| S1PR3 | #1: 5-GCGGCACUUGACAAUGAUCAATT-3 |
|  | #2: 5-CAUCGCUUACAAGGUCAACAUTT-3 |
| FGFR1 | #1: 5-GCACATCCAGTGGCTAAAGCAC-3 |
|  | #2: 5-AGCACCTCCATCTCTTTGTCGG-3 |
| STAT4 | #1: 5-GAAUCAAGUCCAACAGUUATT-3 |
|  | #2: 5-GAACUAAACUAUCAGGUAATT-3 |

**TABLE S5** Primer sequences used for ChIP-qPCR analysis of the S1PR3 promoter

| Binding elements | Sequence | Position | Primer name | Primer sequence |
| --- | --- | --- | --- | --- |
| 1 | TTTTCC | -1967 to -1962 | 1/2 F  1/2 R | CAGAATGCCCACACTCTGGT  GCAAAGCCTCTAGACTTTACTGC |
| 2 | ATTTCC | -1926 to -1921 | 1/2 F  1/2 R | CAGAATGCCCACACTCTGGT  GCAAAGCCTCTAGACTTTACTGC |
| 3 | GGAAAA | -1737 to -1732 | 3/4/5 F  3/4/5 R | CCAGGTTGAGCCAGTATTAG  CAGGCGAACGGGTGCTAAT |
| 4 | GGAAAA | -1730 to -1725 | 3/4/5 F  3/4/5 R | CCAGGTTGAGCCAGTATTAG  CAGGCGAACGGGTGCTAAT |
| 5 | TTTTCC | -1710 to -1705 | 3/4/5 F  3/4/5 R | CCAGGTTGAGCCAGTATTAG  CAGGCGAACGGGTGCTAAT |
| 6 | ATTTCC | -1604 to -1599 | 6 F  6 R | GACACCCACTAGTGCACAC  GAATGCCAGCTCATAAACG |
| 7 | GGAATT | -1287 to -1282 | 7/8 F  7/8 R | CCCACTGAGGACAGGGATC  GCCCAGAAGCCCTGAGGAG |
| 8 | GGAAAC | -1243 to -1238 | 7/8 F  7/8 R | CCCACTGAGGACAGGGATC  GCCCAGAAGCCCTGAGGAG |
| 9 | GTTTCC | -925 to -920 | 9 F  9 R | GGTTGTGACCAATGCCAAAGG  CCCAAGTTTCATGTGGGCCT |
| 10 | TTTTCC | -352 to -347 | 10 F  10 R | CTCCCGCTTCTCTCCTCTCT  GCCATGATTGAGCGAACACAG |
| 11 | AGTTCC | -245 to -240 | 11/12 F  11/12 R | CGCTCAATCATGGCCTTTGG  GCCCCTTTTGTGTGGCAAA |
| 12 | GGAATT | -232 to -227 | 11/12 F  11/12 R | CGCTCAATCATGGCCTTTGG  GCCCCTTTTGTGTGGCAAA |
| 13 | GGAAAA | +44 to +49 | 13 F  13 R | GAGCCCAAGTCTCTGCGTG  GCCGGACAGCGTTTTTGTTT |
